# Quantitative analysis of the TNF-α-induced phosphoproteome reveals AEG-1/MTDH/LYRIC as an IKKβ substrate

**DOI:** 10.1038/ncomms7658

**Published:** 2015-04-07

**Authors:** Ramesh K. Krishnan, Hendrik Nolte, Tianliang Sun, Harmandeep Kaur, Krishnamoorthy Sreenivasan, Mario Looso, Stefan Offermanns, Marcus Krüger, Jakub M. Swiercz

**Affiliations:** 1 Department of Pharmacology, Max-Planck-Institute for Heart and Lung Research, Ludwigstrasse 43, 61231 Bad Nauheim, Germany; 2 Biomolecular Mass Spectrometry, Max-Planck-Institute for Heart and Lung Research, Ludwigstrasse 43, 61231 Bad Nauheim, Germany; 3 Department of Cardiac Development and Remodelling, Max-Planck-Institute for Heart and Lung Research, Ludwigstrasse 43, 61231 Bad Nauheim, Germany; 4 Medical Faculty, University of Frankfurt, Theodor-Stern-Kai 7, 60596 Frankfurt am Main, Germany

## Abstract

The inhibitor of the nuclear factor-κB (IκB) kinase (IKK) complex is a key regulator of the canonical NF-κB signalling cascade and is crucial for fundamental cellular functions, including stress and immune responses. The majority of IKK complex functions are attributed to NF-κB activation; however, there is increasing evidence for NF-κB pathway-independent signalling. Here we combine quantitative mass spectrometry with random forest bioinformatics to dissect the TNF-α-IKKβ-induced phosphoproteome in MCF-7 breast cancer cells. In total, we identify over 20,000 phosphorylation sites, of which ∼1% are regulated up on TNF-α stimulation. We identify various potential novel IKKβ substrates including kinases and regulators of cellular trafficking. Moreover, we show that one of the candidates, AEG-1/MTDH/LYRIC, is directly phosphorylated by IKKβ on serine 298. We provide evidence that IKKβ-mediated AEG-1 phosphorylation is essential for IκBα degradation as well as NF-κB-dependent gene expression and cell proliferation, which correlate with cancer patient survival *in vivo*.

## Abstract (web summary)

Inflammatory cytokines such as TNF-α influence inflammation, apoptosis and tumour development through regulation of the kinase IKKβ. Krishnan and Nolte *et al.* apply quantitative proteomics to identify potential IKKβ targets, and reveal phosphorylation of AEG-1 by IKKβ as a mechanism controlling NF-κB signalling.

## Graphical abstract

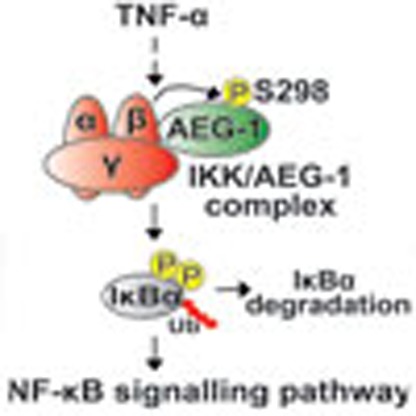

NF-κB activation-mediated gene transcription is critical for physiological and pathological processes including stress responses, apoptosis and tumour development^1^. It has been shown that a broad range of stimuli induce NF-κB activation, which results in the activation of an array of transcriptional programmes^2^. Moreover, NF-κB-dependent transcription is not only tightly controlled by positive and negative regulatory mechanisms but also closely coordinated with other signalling pathways^3^, such as the insulin and MAP kinase signal transduction.

The I-kappa-B kinase (IKK) complex is a crucial activator of NF-κB^4^ and consists of three proteins: IKKα (CHUK), IKKβ and IKKγ (NEMO)^2^. The complex can be activated through the phosphorylation of the key serine residues in the T loops of IKKα and IKKβ by oligomerization-induced autophosphorylation^5^. The activated IKK complex phosphorylates IκB proteins in the conserved destruction box, which leads to their proteasomal degradation^2^. NF-κB subunits are then translocated to the nucleus to regulate the transcription of target genes. There is an increasing evidence for NF-κB-independent IKK signalling and various non-IκB targets were identified for IKKα, β and γ, implicating that their function is not restricted to the regulation of NF-κB-mediated gene expression^1^. Indeed, IKKβ-induced phosphorylation events were shown to negatively regulate proteins involved in apoptosis, inflammation and proliferation control. For example, IKKβ phosphorylates the tumour suppressor Forkhead box protein O3 (FOXO3a) on serine 644 leading to reduced translocation into the nucleus and increased FOXO3a degradation. Moreover, FOXO3a phosphorylation results in cell cycle arrest and enhanced apoptosis by FOXO3a-dependent transcription of genes encoding molecules^1^.

IKKβ is also capable of phosphorylating Hamartin (TSC1), a known repressor of the small GTPase Rheb within mammalian target of rapamycin (mTOR) signalling^6^. IKKβ-dependent phosphorylation of serine 505 interferes with TSC1 function and is required for mTOR activation in response to pro-inflammatory cytokines such as tumour necrosis factor (TNF-α)^6^. Other known phosphorylation targets of IKKβ include MAPK pathway regulators: p105, Dok3 and SNARE protein SNAP23 (ref. 1). In addition, IKKβ influences the development of insulin resistance by phosphorylation of IRS1 (ref. 1).

Although considerable progress has been made in understanding NF-κB pathway-dependent and -independent functions of IKKβ, the recent evidence for new IKKβ targets suggest that many of its functions still remain to be discovered.

To search for new potential substrates of the IKK complex, we study IKKβ-mediated phosphorylation in stable isotope labelling by amino acids in cell culture (SILAC)-labelled breast carcinoma cells. Here we test the effect of the TNF-α stimulation and IKKβ inhibition on the phosphoproteome. High-resolution mass spectrometry and comprehensive bioinformatics enable us to identify a plethora of new proteins being potential novel targets of IKKβ. We show that one of these candidates—Astrocyte-Elevated Gene-1 (AEG-1), also known as Metadherin (MTDH) or lysine-rich CEACAM1 co-isolated (LYRIC) is a direct substrate of IKKβ. On TNF-α activation, IKKβ phosphorylates particular serine residue of AEG-1, which is a crucial step required for downstream cellular effects of TNF-α.

## Results

### Analysis of IKKβ-mediated phosphorylation in MCF-7 cells

**Figure 1:**
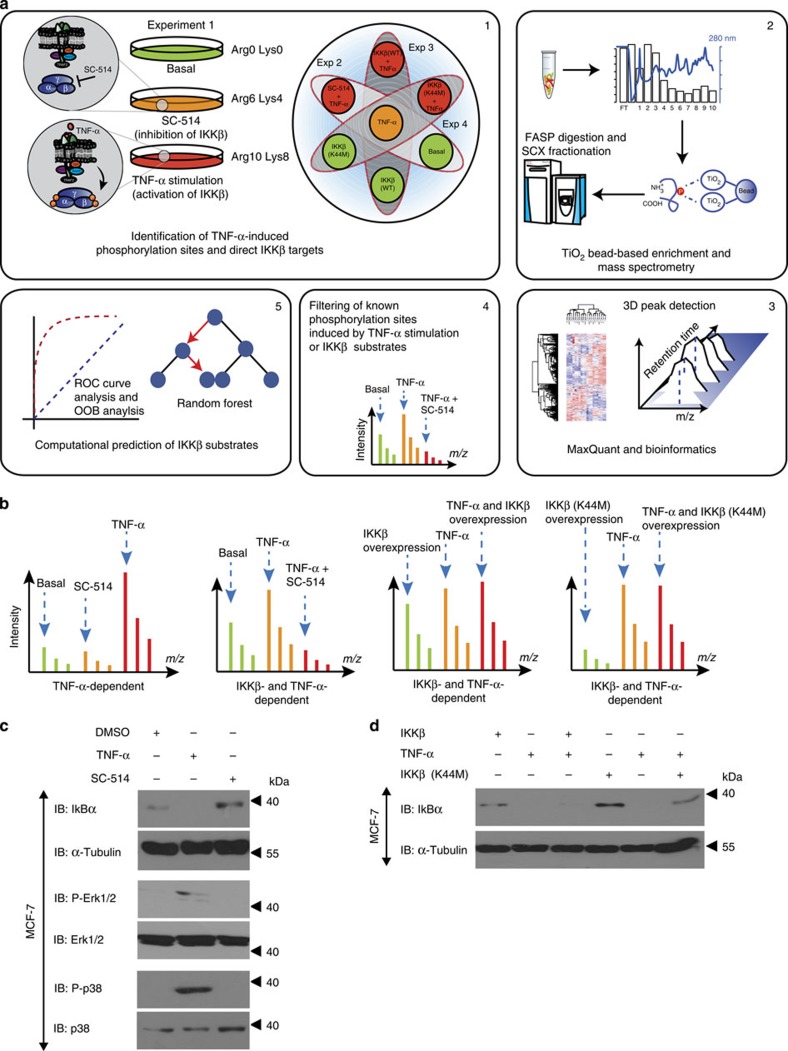
Quantification of the TNF-α/IKKβ-regulated phosphoproteome in MCF-7 cells. (**a**) Tandem mass spectrometry-based schematic workflow of the experimental set-up consisting of four SILAC experimental strategies allowed the quantification of phosphorylation across nine different conditions (see Methods for detailed description). (**b**) Representative SILAC spectra with interpretation (See also Supplementary Fig. 1). (**c**) SILAC-labelled MCF-7 cells were starved and treated with DMSO, TNF-α or SC-514 as indicated. Cells were lysed as described in Methods section. Cell lysates were separated using SDS–PAGE, transferred to a nitrocellulose membrane and protein expression and phosphorylation were detected using indicated antibodies. (**d**) SILAC-labelled MCF-7 cells were transfected with control vector, wild-type (WT) IKKβ or with kinase dead IKKβ (K44M) as indicated. Forty-eight hours after transfection, cells were starved for 12 h and treated either with control (−) or with TNF-α (+). Cells were then lysed, and IκBα and α-tubulin were detected using the respective antibodies.

We used the breast tumour cell line MCF-7 (ref. 7) to investigate phosphorylation events downstream of IKKβ and applied stable isotope labelling by amino acids in cell culture (SILAC) for the accurate quantitation of dynamic phosphopeptides. We combined four ‘double-triple' SILAC labelling experiments on the basis of arginine and lysine labelling. A schematic visualization is shown in Fig. 1a. The nonstimulated cell population (Arg0 and Lys0) was compared with cells stimulated with TNF-α for 10 min. Experiment (Exp.) 1 was used as a control for investigating the changes in cellular phosphorylation after treatment with a selective IKKβ inhibitor (SC-514; Arg6 and Lys4) or TNF-α (Arg10 and Lys8). To gain more insight into TNF-α-dependent IKKβ activity, we treated the Arg10, Lys8 cell population with SC-514 before TNF-α stimulation (Exp. 2). Moreover, we transfected Arg0, Lys0 and Arg10, Lys8 populations with a wild-type or a kinase dead mutant of IKKβ (K44M; Exp. 3 and Exp. 4, respectively). In all the experiments, direct comparison of cell populations was achieved by using TNF-α-stimulated cells (labelled with Arg6/Lys4 or Arg10, Lys8) as a reference. Thus, SILAC multiplexing allowed us to directly compare nine distinct conditions with each other (Fig. 1a).

We mixed the related cell populations (Exp. 1–Exp. 4) and the extracted proteins were in solution digested with trypsin. Next, the peptides were separated using strong cation exchange chromatography (SCX) and the phosphopeptides were enriched with titaniumdioxide (TiO_2_) beads. All samples were analysed using nanoLC-MS/MS on the QExactive mass spectrometer and raw spectra were processed using MaxQuant (Fig. 1a).

In total, we identified over 20,000 phosphorylation sites from 4,616 proteins (Supplementary Data 1). Mass deviation of less than 2 p.p.m. for more than 95% of all identified peptides demonstrated high mass accuracy and reproducibility between two independent biological replicates was determined by Pearson correlation and ranged between 0.65 and 0.83 for all four experimental set-ups (Supplementary Fig. 1a,b). To test the expression changes on TNF-α stimulation, we used a class of unmodified peptides for protein quantification and observed SILAC ratio close to 1:1 for over 97% of all quantified proteins (6,024, 81%; Supplementary Fig. 1c,d; Supplementary Data 2). This indicated that phosphorylation changes are not due to differences in protein abundance and that normalization is not required. In total, we identified 254 phosphorylation sites that were significantly regulated on TNF-α stimulation. Among those, 193 phosphopeptides were upregulated with a fold change >1.5 at a false discovery rate (FDR) <0.05. Notably, the combined treatment with TNF-α and SC-514 resulted in a clear inhibition (>90%) of 77 TNF-α-dependent phosphorylation sites (Exp. 2, Supplementary Data 1). The overlap to the SC-514 inhibition compared with the overexpression of the IKKβ kinase dead mutant (K44M; Exp. 4) was ∼75%. This indicates that the experimental set-up is sufficient to analyse IKKβ-dependent signal transduction.

A schematic overview of possible SILAC ratio combinations is depicted in Fig. 1b. For example, Exp. 2 reveals TNF-α dependency under the Arg6, Lys4-labelled condition, whereas the Arg10, Lys8 condition allows for the assessment of IKKβ-specific downstream phosphorylation sites. Exp. 3 and 4 allowed us to understand basal, IKKβ-dependent phosphorylation in MCF-7 cells and the conditions for mechanisms underlying the increase in phosphorylation on TNF-α stimulation in cells overexpressing IKKβ.

In response to TNF-α stimulation, IκBα proteins are phosphorylated and as a consequence degraded, thus providing a positive control for our data set. As an example, we have identified the phosphorylation sites S32, S36 of the NF-kappa-B inhibitor alpha (IκBα) protein with a 22-fold upregulation on TNF-α stimulation, whereas treatment of SC-514 showed a significant downregulation (53%). Similarly, one of the known substrates of IKKβ, serine 312 of IRS1, was found to be upregulated fivefold on TNF-α stimulation and downregulated after treatment with SC-514. We also saw a downregulation by 80% of the IKKβ autophosphorylation site serine 698 on SC-514 treatment. Of note, we observed a clear activation of wild-type IKKβ on TNF-α stimulation, whereas the IKKβ kinase dead mutant did not show any activation in response to TNF-α. In a parallel experiment, SILAC-labelled MCF-7 cell lysates, used for mass spectrometry analysis, were subjected to immunoblotting and we observed a moderate degradation of IκBα in control MCF-7 cells, suggesting a basal activity of the IKK complex. We were able to see an increased degradation of inhibitor of the nuclear factor-κB (IκBα) on TNF-α stimulation; however, this effect was abolished on SC-514 treatment or on the overexpression of the kinase dead IKKβ (K44M), thus proving the basal functionality of the experimental conditions (Fig. 1c,d). In this way, our global phosphopeptide analysis covered a variety of TNF-α- and IKKβ-mediated phosphorylation as well as the majority of all known TNF-α pathway member events in MCF-7 breast cancer cells (Supplementary Fig. 2). The novel inhibitory effect of SC-514 was demonstrated by the known substrates used subsequently to train random forest predictor to obtain a ranked list of potential IKKβ substrates (Supplementary Data 1).

### Random forest-based prediction of potential IKKβ substrates

**Figure 2:**
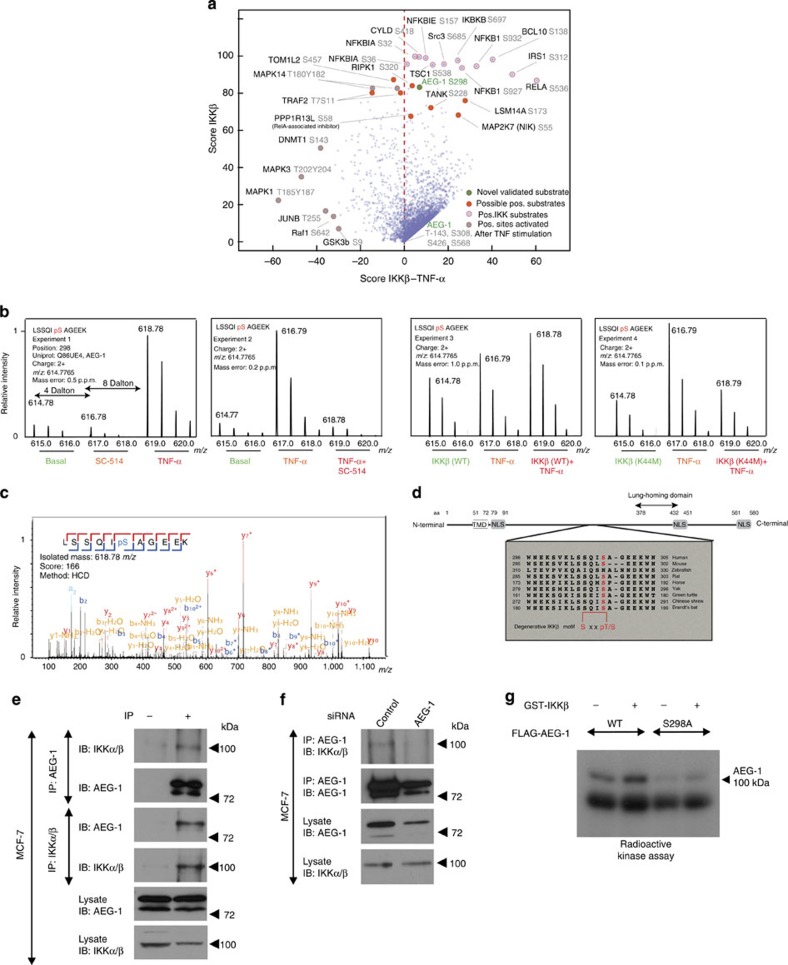
Identification of serine 298 of protein AEG-1 as a direct target of IKKβ. (**a**) Shown are the results that were obtained from the random forest analysis using known IKKβ- and TNF-α-mediated phosphorylation sites. High scores of known positive hits used in IKKβ-dependent phosphorylation category showed the applicability of this ensemble-learning machine. Furthermore, known TNF-α phosphorylation sites on proteins such as JNK, Raf1, JunB and Erk1/2 fall into the bottom left corner of the scatter plot, indicating a high TNF-α score compared with predicted targets for IKKβ. Score of AEG-1 at serine 298 was shown in green (see also Supplementary Figs 2 and 3). (**b**) Representative MS SILAC spectra for each experiment (Exp. 1–4). Andromeda Score for corresponding MS/MS spectra and mass deviation in p.p.m. is given. See main text for detailed interpretation of these changes. (**c**) MS/MS spectra for localization and identification of S298 using HCD fragmentation and accurate mass measurement in the Orbitrap (QExactive). (**d**) Schematic representation of the domain structure of protein AEG-1 and sequence homology of the region surrounding serine 298 between different species. (**e**) Endogenous protein AEG-1 or IKKα/β were immunoprecipitated from MCF-7 cells. Protein–protein interactions were visualized using respective antibodies. (**f**) MCF-7 cells were transfected with control siRNA or siRNA directed against AEG-1. Forty-eight hours after transfection, AEG-1 was immunoprecipitated from the cell lysates and protein–protein interactions were visualized by immunoblotting with the respective antibodies. (**g**) FLAG-tagged WT or S298A AEG-1 was overexpressed in HEK293 cells. After 48 h of incubation, overnight serum-starved cells were lysed and immunoprecipitated using anti-FLAG affinity gel. Active recombinant IKKβ was incubated with the immunoprecipitated complex in the presence of γ^32^-ATP and kinase buffer for 30 min. At the end of the kinase reaction, samples were boiled in Laemmlli buffer and separated using SDS–PAGE and exposed to X-ray film.

To gain a more systematic view of the potential target proteins in TNF-α-dependent IKKβ activity, we used a random forest-based approach to identify substrates of IKKβ under physiological conditions (Figs 1a, 4 and 5). Random forest is an ensemble-learning algorithm for the classification of large-scale data sets^8^. We quantified 25 known TNF-α-dependent phosphorylation sites and 11 known substrates of IKKβ in all the experiments of our data set and used those candidates for sorting to create positive data set (Supplementary Data 1; Supplementary Fig. 3). After the generation of the random forest predictor, we analysed the complete phosphopeptide data set to calculate the probability of IKKβ and TNF-α classifiers. Thus, we were able to calculate a score for each phosphorylation site on the basis of our quantitative phosphoproteome analysis containing nine distinct conditions, and we found relative high scores for all identified IKKβ substrates and TNF-α responders (Supplementary Data 3). To compare and evaluate both predictors, we plotted the delta IKKβ-TNF-α score versus the IKKβ score (Fig. 2a). We obtained a higher or almost equal score for IKKβ compared with TNF-α classification and identified five novel potential IKKβ substrates (Table 1). TOM1 like protein 2 (TOM1L2) was suggested to play several roles in various cellular processes including protein transport and mitosis^9^,^10^. We observed a TNF-α-mediated, 10-fold upregulation of a phosphorylated serine at position 457, which was then markedly reduced after IKKβ inhibition. Moreover, increased wild-type IKKβ expression led to an upregulated TOM1L2 phosphorylation, whereas the kinase-inactive IKKβ had no effect. Another candidate showing increased phosphorylation at serine 320 on TNF-α stimulation was the receptor-interacting serine/threonine protein kinase 1 (RIPK1)^11^. This kinase is known to be critically involved in the activation of necroptosis in a TNF-α-dependent manner. Even though it has been shown that RIPK1 is phosphorylated at serine 320 (ref. 12), the kinase responsible for the phosphorylation and its functional significances are unknown. Our data implicated that TNF-α-dependent IKKβ-mediated phosphorylation of RIPK1 may represent a novel *in vivo* regulatory mechanism. Furthermore, we detected a IKKβ-dependent phosphorylation of autophagy-related protein 2 homologue B (ATG2B) on serine 497 and synaptotagmin-like protein 2 (SYTL2) on serine 649. ATG2B is involved in the regulation of the autophagosome formation and lipid droplet morphology, whereas SYTL2 acts as a RAB27A effector protein and plays a role in cytotoxic granule exocytosis in lymphocytes^13^,^14^. In both cases, our data suggested a novel regulatory role of TNF-α/IKKβ signalling in those processes. Taken together, the random forest algorithm allowed us to identify several novel potential IKKβ-dependent proteins.

### AEG-1 is a novel potential substrate of IKKβ

Another candidate that we identified with a high IKKβ score (Table 1) is protein AEG-1 (LYRIC/MTDH)^15^,^16^,^17^,^18^. AEG-1 is a transmembrane protein that shares no homology to other known genes^15^. Various studies have localized AEG-1 to the cell membrane, nucleus and endoplasmic reticulum^15^,^16^,^19^. AEG-1 has been linked to the regulation of cell proliferation, survival and motility by controlling the MAPK pathway and via activation of NF-κB^20^,^21^,^22^. Clinical data show a close correlation between the expression of AEG-1 and poor survival prognosis in different types of tumour^23^,^24^,^25^. We identified a novel regulatory serine phosphorylation site at the position 298 with a fourfold increase on TNF-α stimulation (Fig. 2a and Supplementary Data 2). Selected SILAC pairs and the tandem mass spectrometry (MS/MS) spectra of the phosphorylated S298 are shown in (Fig. 2b,c). Moreover, overexpression of wild-type IKKβ also increased serine 298 phosphorylation in control and TNF-α-stimulated cells, whereas kinase dead IKKβ mutant did not show any regulation of S298 (Fig. 2b,c and Supplementary Data 1 and 2), indicating that the kinase activity of IKKβ is crucial for AEG-1 phosphorylation and IKKα is not necessary for phosphorylation of AEG-1. Other phosphorylation sites of AEG-1 (T143, S308, S426 and S568; Fig. 2a) showed the score close to zero for IKKβ and TNF-α stimulation predictors, highlighting the importance of S298 in TNF-α-mediated signalling.

AEG-1 shares no homology to the identified domains; however, its sequence includes several potential nuclear localization signals (Fig. 2d). Serine 298, which we found to be regulated in dependence of IKKβ activity, is localized within the degenerative IKKβ recognition motif SXXpS/T (Fig. 2d). Sequence analyses showed that serine 298 is located in the region that is highly conserved across several species (Fig. 2d); unfortunately, the biological role of this region is unknown, making it impossible to predict the impact of IKKβ-mediated phosphorylation of serine 298 on AEG-1 activity.

### IKKβ interacts with and directly phosphorylates AEG-1

The specificity of the global phosphopeptide screen does not allow for understanding the differentiation between direct and indirect kinase targets. In order to test whether AEG-1 can directly be phosphorylated by IKKβ, we took advantage of the findings showing the association of kinases with their targets^26^. Therefore, we tested the interaction between endogenous IKKβ and AEG-1 in MCF-7 cells using immunoprecipitation. Indeed, we observed the stable specific (as controlled by short interfering RNA (siRNA)-mediated knockdown of AEG-1) interaction between endogenous AEG-1 and IKKβ in MCF-7 cells (Fig. 2e,f), suggesting the possibility of direct phosphorylation of AEG-1 via IKKβ. In order to further support the hypothesis of the direct interaction and phosphorylation of AEG-1 by IKKβ, we performed radioactive *in vitro* kinase assay using P^32^-ATP. We observed that AEG-1 but not its serine 298 mutant is directly phosphorylated by IKKβ (Fig. 2g), thus establishing AEG-1 as a novel direct target of IKKβ.

### AEG-1 regulates IκBα degradation in an IKKβ-dependent way

AEG-1 has been shown to promote various cellular processes including cell migration, gene expression and cell proliferation of various cancer types, such as hepatocellular carcinoma (HCC), oesophageal squamous cell carcinoma, and breast and prostate cancers^17^,^27^,^28^,^29^,^30^,^31^,^32^. Therefore, we performed a series of experiments aiming at pinpointing the role of IKKβ-mediated phosphorylation of serine 298 of AEG-1 on its cellular functions.

**Figure 3:**
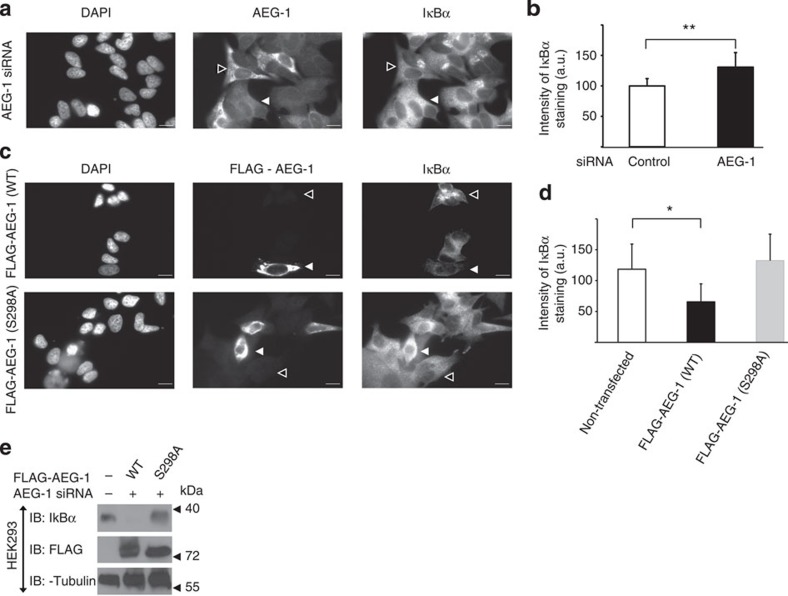
IKKβ-mediated IκBα degradation is blocked by S298A AEG-1. (**a**) MCF-7 cells were transfected with AEG-1 siRNA. Forty-eight hours later, cells were starved for 12 h and fixed in 4% PFA for 15 min at room temperature. Cells were immunostained using anti-AEG-1 and anti-IκBα antibodies. (**b**) Cells stained in **a** were imaged using epi-fluorescence microscope and cell borders were marked using ImageJ. Staining intensity was analysed using ImageJ and normalized to the cell area. Plots show the mean values of at least nine cells per condition from two independent experiments±s.d. Data were analysed by Student's *t*-test, **P<0.005. (**c**) MCF-7 cells were transfected with WT, FLAG-tagged AEG-1 or its FLAG-tagged serine-to-alanine mutant (S298A). Forty-eight hours after transfection, cells were fixed and stained using anti-FLAG or anti-IκBα. Cell nuclei were visualized using Hoechst 33342. (**d**) Intensity of MCF-7 stained in **c** was analysed as described above. Plots show average values of at least nine cells per condition from two independent experiments±s.d. Data were analysed by Student's *t*-test, *P<0.05. (**b**). Scale bar, 100 μm. (**e**) HEK293 cells were transfected with control siRNA or with siRNA against AEG-1 and rescued with either WT AEG-1 or its S298A mutant (as indicated). Cells were lysed and proteins were detected with the indicated antibodies.

Next, we analysed the role of serine 298 of AEG-1 in the activation of NF-κB. It has been proposed that AEG-1 mediates IκBα degradation^33^. Indeed, IκBα staining of MCF-7 cells transfected with siRNA against AEG-1 revealed that AEG-1 knockdown resulted in the significant inhibition of IκBα degradation as compared with nontransfected cells (Fig. 3a,b). On the contrary, the overexpression of wild-type AEG-1 in MCF-7 cells resulted in the rapid degradation of IκBα (Fig. 3c,d). Interestingly, overexpression of mutated AEG-1 (S298A) almost completely blocked IκBα degradation in MCF-7 cells (Fig. 3c,d), suggesting that phosphorylation of serine 298 of AEG-1 is crucial for its function in the activation of NF-κB. In order to further understand the role of serine 298 in the activation of NF-κB, we have analysed levels and post-translational modifications of IκBα in HEK293 cells depleted of AEG-1 and rescued with wild-type AEG-1 or its S298A mutant. We observed that the expression of wild-type AEG-1 resulted in the complete degradation of IκBα, whereas no degradation was detected in cells transfected with phosphorylation-insensitive S298A AEG-1 (Fig. 3e).

IκBα becomes phosphorylated and ubiquitinated on its way to degradation. To learn more about the role of AEG-1 phosphorylation in IκBα degradation, we decided to analyse post-translational modifications of IκBα in cells transfected with either wild-type AEG-1 or its S298A mutant (Supplementary Fig. 4a,b) and found that both IκBα phosphorylation and ubiquitination were strongly decreased in the cells expressing phosphorylation-insensitive AEG-1(S298A; Supplementary Fig. 4a,b), this finding once again underscores the importance of IKKβ-mediated phosphorylation of AEG-1 for IκBα degradation.

Finally, we attempted to analyse the protein–protein interactions involved in AEG-1-mediated degradation of IκBα. We employed HEK293 cells and tested the possibility that AEG-1 forms complexes with both IKKβ and IκBα. In agreement with our previous data from MCF-7 cells (Fig. 2e,f), we could also observe an interaction between AEG-1 and IKKβ in HEK293 cells (Supplementary Fig. 4c). Interestingly, mutation of serine 298 to alanine strongly increased the interaction between AEG-1 and IKKβ (Supplementary Fig. 4c). Moreover, using overexpressed AEG-1, we were able to detect an IKKβ/AEG-1/IκBα complex, whereas no complex was detected in cells transfected with mutated AEG-1 (Supplementary Fig. 4a). Taken together, these data imply that the interaction between AEG-1 and IKKβ, and the subsequent phosphorylation of AEG-1 on serine 298, are a prerequisite for the association of AEG-1 with IκBα and its subsequent degradation.

### AEG-1 phosphorylation mediates TNF-α-induced gene expression

**Figure 4:**
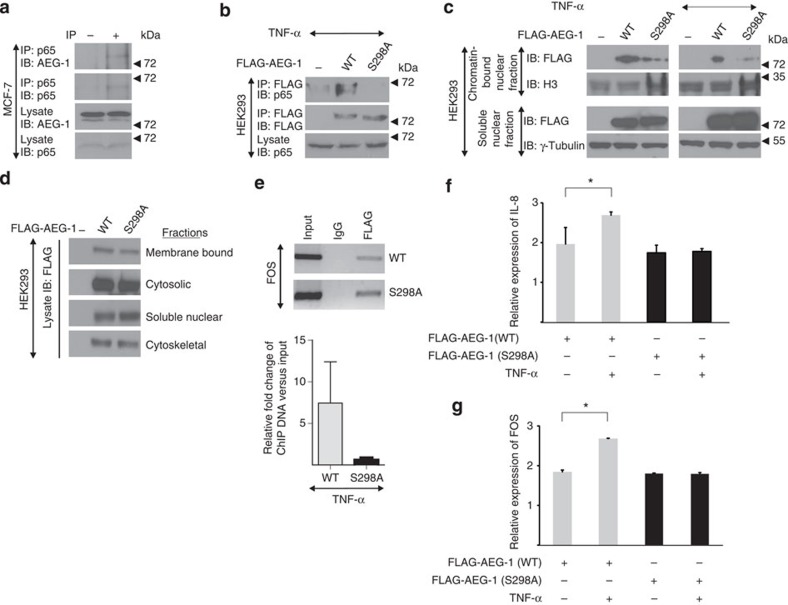
Serine 298 of AEG-1 is required for NF-κB-mediated gene regulation. (**a**) p65 was immunoprecipitated from MCF-7 cells and the precipitates were immunoblotted using anti-AEG-1 and anti-p65 antibodies. (**b**) HEK293 cells were transfected with control, FLAG-tagged WT or S298A mutant of AEG-1. After 48 h, cells were serum-starved and stimulated with TNF-α (10 ng ml^−1^). AEG-1 was immunoprecipitated using anti-FLAG agarose. P65 and AEG-1 were visualized using their specific antibodies. (**c**) HEK293 cells were transfected as described above (**b**) and treated with either control (−) or TNF-α (+). Cellular fractions were prepared as described in the Methods section. Proteins were visualized in the soluble nuclear and chromatin-bound fractions using their respective antibodies. (**d**) HEK293 cells were transfected as described above. Forty-eight hours after transfection, cellular fractions were prepared using the fractionation kit (Thermo) according to the manufacturer's instructions. (**e**) MCF-7 cells were transfected with the FLAG-tagged WT or the S298A mutant of AEG-1. After 48 h, cells were serum-starved and stimulated with TNF-α (10 ng ml^−1^) for 6 h. Cells were fixed using (1%) formaldehyde, crosslinked with DNA and precipitated using anti-FLAG affinity gel. Eluted DNA from the precipitates was amplified by using primers binding to FOS promoter (upper panel) and quantified by real-time PCR as described in the Methods section. Shown are the mean values from three independent experiments±s.d. Data were analysed by Student's *t*-test, *P<0.05. (**f**,**g**) MCF-7 cells were transfected as described, treated with control (−) or TNF-α (+) and RNA was isolated. Expression of NF-κB-regulated genes, IL-8 (**e**) and FOS, (**f**) was determined. Data are expressed as mean values±s.d. from at least duplicates. Data were analysed by Student's *t*-test, *P<0.05.

AEG-1 was shown to directly interact and translocate to the nucleus with the p65 subunit of the p50/p65 complex of NF-κB^33^. Indeed, we observed that AEG-1 interacts with p65 in MCF-7 cells (Fig. 4a). As AEG-1/p65 can be translocated to the nucleus on TNF-α stimulation and this process is crucial for the p65-mediated transcription^33^, we tested the interaction between S298 mutant and p65 in the nucleus in TNF-α-stimulated cells. In the transfected and TNF-α-treated HEK293 cells, we observed that wild-type AEG-1 interacts with p65; this interaction is lost when serine 298 is mutated to alanine (Fig. 4b), suggesting that IKKβ-mediated phosphorylation of AEG-1 is a prerequisite for AEG-1-p65 interaction in the nucleus. Surprisingly, we observed that mutated AEG-1 translocates to the nucleus comparably to the wild-type protein, albeit its chromatin-bound fraction is markedly decreased (Fig. 4c). In addition, we studied the localization of AEG-1 in other cellular compartments and again observed that both AEG-1 and its S298A mutant localize in a similar way (Fig. 4d), thus showing that IKKβ-mediated phosphorylation of AEG-1 is dispensable for its cellular localization.

AEG-1 mediates interaction between p65, CBP and the other basal transcriptional components, facilitating NF-κB-driven gene transcription^34^. To support these findings, we analysed FOS-promoter-bound fraction of AEG-1 in MCF-7 cells overexpressing wild-type or point-mutated AEG-1 (S298A). Using a nonquantitative and quantitative PCR, we found that the mutation of serine 298 disturbed AEG-1 binding to DNA (Fig. 4e). These findings suggested that the phosphorylation of serine 298 is critical for interaction with p65 and also important for binding to DNA. These findings provide an explanation for the role of AEG-1 phosphorylation in TNF-α-mediated gene expression. Activation of NF-κB by TNF-α represents a crucial step leading to the expression of a variety of genes^35^; in addition, AEG-1 has been shown to induce NF-κB-mediated expression of the intracellular adhesion molecule-3 and -2, selectin E and L, toll-like receptor-4, FOS, JUN and interleukin-8 (IL-8)^33^. Since our data indicated that the phosphorylation of AEG-1 via IKKβ may be a crucial step in this process, we therefore analysed the effects of AEG-1 S298 mutation on the expression of FOS and IL-8 in HEK293 cells and observed that TNF-α stimulation of AEG-1-transfected cells resulted in the significantly increased expression of both IL-8 and FOS (Fig. 4f,g). This effect was completely abolished by overexpression of mutated AEG-1 (S298A; Fig. 4f,g). In addition, we investigated the impact of the IKKβ-dependent phosphorylation of AEG-1 on 4T1 cells stably transfected with wild-type or mutated AEG-1. In agreement with our previous data, we observed that the mutation of serine 298 completely abolished effects of AEG-1 overexpression on FOS expression, thus strengthening the notion that IKKβ-mediated AEG-1 phosphorylation is necessary for TNF-α-driven gene transcription downstream of NF-κB (Supplementary Fig. 5a).

### AEG-1 phosphorylation in cell proliferation and survival

**Figure 5:**
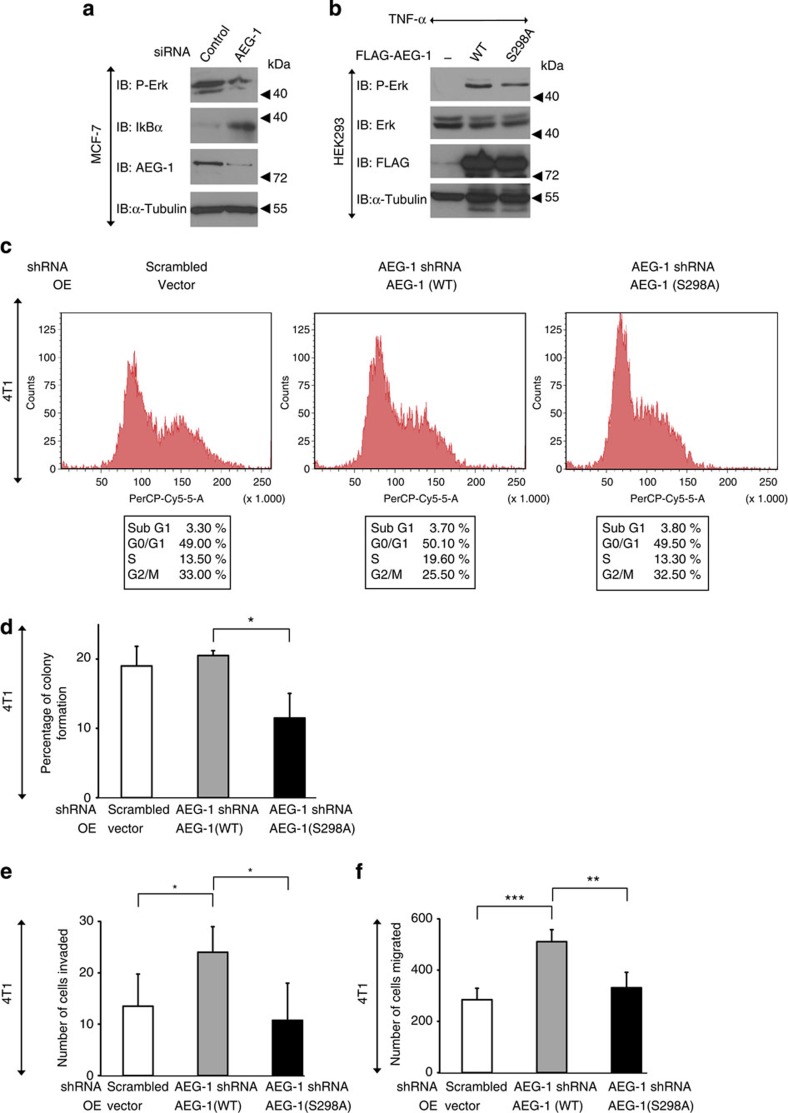
Serine 298 of AEG-1 is necessary for cell proliferation and migration. (**a**) siRNAs targeting AEG-1 were transfected into MCF-7 cells. Cell lysates were immunoblotted with specific antibodies. (**b**) HEK293 cells were transfected with control, WT AEG-1 or its mutant (S298A). Cells were starved, treated with TNF-α and cell lysates were immunoblotted as described above. (**c**–**f**) 4T1 cells were stably transfected control shRNA or shRNA directed against AEG-1 together with WT or mutant (S298A) AEG-1. (**c**) Cells were trypsinized and cell cycle was analysed with FACS. (**d**) Cells were seeded to the density of 100 and allowed to grow for 5 days. Cells were then stained and colonies containing more than 50 cells were counted. Shown are the mean values from two independent experiments±s.d. Data were analysed by Student's *t*-test, *P<0.05. (**e**) Cells were trypsinized, counted and 10,000 cells in RPMI containing 0.5% FBS were seeded into the upper chamber of a transwell plate. Cells were allowed to migrate for 24 h. (**f**) Overall, 15,000 cells were seeded on top of the matrigel invasion plate and allowed to invade for 24 h. Migrated/invaded cells were then fixed, stained and counted as described in Methods. Shown are the mean values±s.d. from tetraplicates. Data were analysed by Student's *t*-test, **P<0.005, ***P<0.0005.

Next, we performed an analysis of the impact of IKKβ-mediated AEG-1 phosphorylation on other known biological functions. AEG-1 has been associated with the Wnt/β-catenin pathway in several cancer conditions. In HCC cells AEG-1 activates Erk1/2, leading to an upregulation of LEF1 expression resulting in the activation of Wnt^27^. In addition, it has been shown that AEG-1 can activate mitogen-activated kinases by increasing Erk1/2 activity^27^. AEG-1 knockdown results in the decreased phosphorylation of Erk1/2 in MCF-7 cells (Fig. 5a) hinting at a relevant function of AEG-1 in our cellular system. Therefore, we tested whether AEG-1 can also affect Erk1/2 phosphorylation in HEK293 cells transfected with either wild-type or mutated AEG-1 (S298A), and found that the overexpression of AEG-1 resulted in the activation of Erk1/2 (Fig. 5b). This effect was partially attenuated in cells transfected with the S298A mutant of AEG-1 (Fig. 5b), suggesting that IKKβ-mediated phosphorylation of AEG-1 is an important but not indispensable step in AEG-1-mediated Erk1/2 phosphorylation.

We also further investigated the effects of serine 298 on cell proliferation. First, we studied the cell cycle distribution in stable 4T1 cells using fluorescence-activated cell sorting (FACS)^36^ and found that overexpression of wild-type AEG-1 markedly increased the entry of cells in the S-phase, whereas mutation of serine 298 abolished this effect (Fig. 5c). In addition, we evaluated the cell cycle distribution in MCF-7 cells stably transfected with wild-type or the serine mutant of AEG-1 and observed similar effects on the cell cycle (Supplementary Fig. 5e), thus indicating that IKKβ-mediated phosphorylation is crucial for AEG-1-mediated gene expression and proliferation. Next, we employed a colony formation assay to analyse cell proliferation in stably transfected 4T1 cells and observed that the mutation of serine 298 strongly abolished cell proliferation (Fig. 5d). We also analysed the proliferation of MCF-7 cells using a label-free, real-time measurement with the xCELLigence system. We noted that transfection of MCF-7 cells with the S298A mutant of AEG-1 significantly decreased cell proliferation (Supplementary Fig. 5b). In addition, we performed a FACS analysis of the proliferation marker Ki-67 in transfected MCF-7 cells. Again, we observed that overexpression of S298A markedly decreased cell proliferation (Supplementary Fig. 5c), underlining an important role of IKKβ phosphorylation of AEG-1 in TNF-α-mediated cell survival.

### Serine 298 of AEG-1 is involved in cell migration

TNF-α is known to enhance cell migration and metastasis of various cancer types through different mechanisms, including the NF-κB-dependent induction of the chemokine receptor CXCR4, upregulation of LOX-1 or activation of matrix metaloproteinases^37^,^38^. In addition, AEG-1 was also shown to enhance the motility and invasive properties of different types of tumours. Our previous results showed that AEG-1 is involved in various NF-κB-mediated signalling events; therefore, we checked whether IKKβ-phosphorylation of AEG-1 is important for an increase in cell migration. By performing a transwell assay, we found that overexpression of the AEG-1 mutant (S298A) significantly decreased cell migration (Supplementary Fig. 5d). Moreover, we tested the effects of the serine 298 mutation on the migration of stably transfected 4T1 cells and again observed that IKKβ-mediated phosphorylation of AEG-1 is crucial for this process (Fig. 5e). Finally, we tested the role of serine 298 on cell invasion. In agreement with the migration data, we observed that 4T1 cell invasion was strongly impaired in cells carrying mutated AEG-1 (Fig. 5f), thus strengthening the hypothesis that IKKβ-mediated phosphorylation of AEG-1 is at least in part responsible for NF-κB-dependent cell migration and invasion.

### Phosphorylation of AEG-1 correlates with patient prognosis

**Figure 6:**
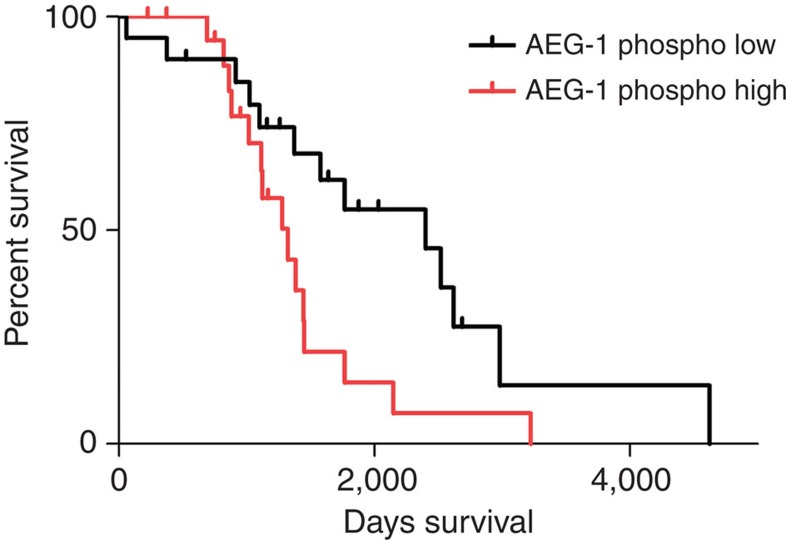
Role of AEG-1 phosphorylation in tumour growth *in vivo*. Kaplan–Meier graph representing the survival of patients dependent on their AEG-1 phosphorylation level. High phosphorylation levels (red, *n*=20) indicate a significantly lower survival rate (log-rank test, *P*=0.0334) than low AEG-1 phosphorylation levels (black, *n*=20).

AEG-1 expression levels were correlated with patient prognosis and metastases in various studies on ovarian cancer^39^,^40^,^41^. In order to determine whether IKKβ-mediated phosphorylation of the serine 298 of AEG-1 in human ovarian cancer influences the progression of the disease, we evaluated a proteomics/phosphoproteomics data set containing detailed patient information^42^. Strikingly, we found that high levels of AEG-1 phosphorylation significantly correlated with shorter survival as compared with patients showing low levels of IKKβ-phosphorylated AEG-1 (Fig. 6). This indicates that IKKβ-mediated phosphorylation of AEG-1 is a crucial factor for tumour growth *in vivo* and that S298 phosphorylation levels can provide additional information on patient prognosis.

**Figure 7:**
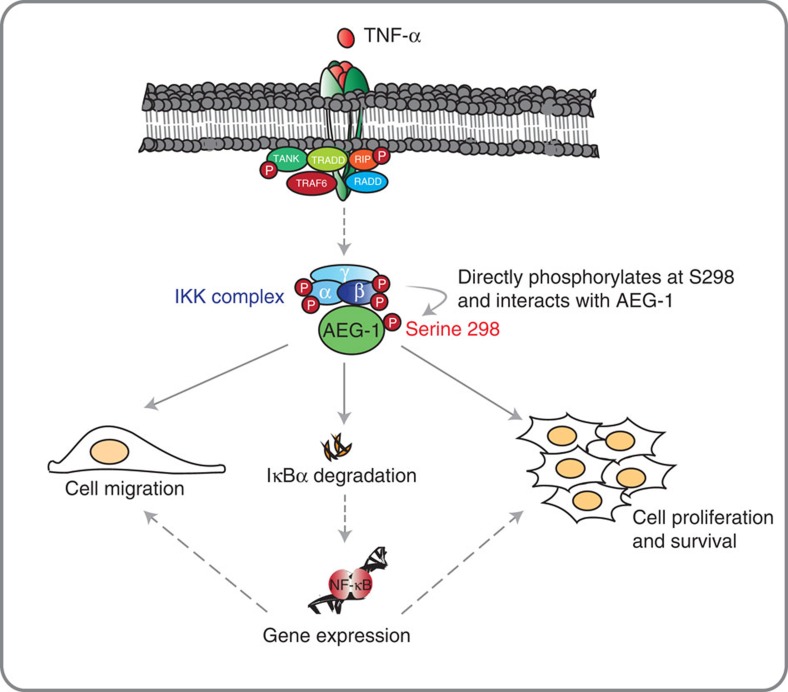
Schematic representation of the significance of AEG-1 serine 298. TNF-α stimulation results in the activation of the IKK complex including IKKβ. IKKβ directly interacts and phosphorylates AEG-1 at serine 298. This step is necessary for IκBα degradation and NF-κB-mediated gene expression. IKKβ-mediated phosphorylation also plays a role in TNF-α-induced cell proliferation and survival as well as cell migration.

Taken together, our data imply that on activation of the IKK complex by TNF-α, IKKβ, phosphorylates serine 298 of the protein AEG-1. This step is crucial for IκBα degradation, the interaction of AEG-1 with p65, the subsequent activation of gene expression downstream of NF-κB and, ultimately, cell proliferation (Fig. 7).

## Discussion

One of the major functions of the TNF-α pathway is the regulation of gene expression through IκBα-kinase-complex (IKK complex)-mediated degradation of IκBα and the subsequent activation of the transcription factor NF-κB^4^. Although regulation of IκBα degradation is believed to be the major cellular function of the IKK complex members, there is growing evidence that the IKK complex has additional functions and modulates other cellular processes, including insulin signal transduction^1^. Here we employed high-resolution mass spectrometry-based quantitative phosphoproteomics to better understand signalling events downstream of IKKβ in breast cancer cells. Using SILAC labelling experiments and Random forest predictions, we were able to validate a data set of TNF-α-induced and IKKβ-dependent phosphorylation sites that are only partially known, presenting a resource for hypothesis-driven investigations on other signalling events downstream of IKKβ. Our data set was validated by the presence of various, already-known IKKβ-dependent phosphorylation sites including IκBα, BCL10 and IRS1 (ref. 1). Interestingly, our analysis revealed that some of the already-known IKKβ targets, including MEKK and ASK1, were not found to be phosphorylated by IKKβ in our experiment. This is intriguing, since the expression of at least some of those proteins was reported in MCF-7 cells^43^. Our finding may implicate that those proteins are not phosphorylated by IKKβ in breast cancer cells. Alternatively, these proteins may be too less abundant or, because of the specific conditions of our experiments, might have escaped our analysis.

At the same time, however, we discovered a variety of novel phosphopeptides, regulated by IKKβ. For example, we observed that IKKβ might be able to phosphorylate the mitogen-activated protein kinase kinase kinase 14 (MAP3K14, also known as NIK). NIK is believed to be an exclusive target for IKKα (ref. 1), and our findings imply a novel crosstalk between the canonical and noncanonical pathways in breast cancers.

We also identified the RIPK1 as a possible IKKβ substrate. Phosphorylation of RIPK1, which transduces inflammatory and cell-death signals (necroptosis)^44^, was previously extensively studied in the context of its autophosphorylation^12^. Interestingly, serine 320, which we identified as a phosphorylation site of RIPK1 in MCF-7 cells, was already reported to be phosphorylated in HEK293T cells. RIPK1 immunoprecipitated from HEK293T cells was found to be stably phosphorylated on serine 320 and it has been shown that the S320-phosphorylated kinase is fully active and able to autophosphorylate *in vitro*. These findings show that phosphorylation of serine 320 does not inhibit RIPK1 kinase activity. We found that serine 320 is strongly phosphorylated in dependence on the TNF-α-IKKβ pathway. Stable phosphorylation in HEK293T cells and regulated phosphorylation in MCF-7 cells are only seemingly contrary, since a basal activity of IKKβ has been reported in cultured cell lines^45^,^46^. The role of serine 320 phosphorylation in RIPK1 functions remains unknown; we can only speculate that, since serine 320-phosphorylated RIPK1 is catalytically active, IKKβ-mediated phosphorylation may be a prerequisite for activation or may regulate cellular localization of RIPK1 *in vivo*.

Similar to serine 320 of RIPK1, phosphorylation of ATG2B on serine 497 has already been reported^47^. Serine 497 is phosphorylated in the liver; however, the responsible kinase or the role of this phosphorylation in ATG2B function remains elusive. Our findings, showing that this residue is phosphorylated in dependence on the TNF-α/IKKβ pathway in breast cancer cells, together with the fact that ATG2B is one of the major regulators of the autophagosome formation and distribution of lipid droplets^14^, suggest a novel IKKβ-dependent regulatory step in these processes under physiological or pathophysiological conditions.

Two other IKKβ-dependent phosphorylation sites we identified, seine 457 of TOM1L2 and serine 649 of SYTL2, are completely novel. Interestingly, both, SYTL2 and TOM1L2, are involved in various forms of cellular trafficking. SYTL2 is an effector of the small GTPase Rab27 and was found to participate in the cytotoxic granule secretion and regulation of localization of proteins to the apical cell surface^48^,^49^, whereas TOM1L2 is related to post-Golgi protein trafficking^10^. The fact that both proteins are strongly phosphorylated after activation of the TNF-α-IKKβ pathway may point to a novel regulatory role of IKKβ in cellular trafficking.

Another novel IKKβ substrate we identified is the protein AEG-1/MTDH/LYRIC, previously shown to localize to different cellular compartments, including the cell membrane^22^,^50^. On the cellular level, AEG-1 was proposed to regulate multiple pathways including the PI3K/AKT pathway, the NF-κB pathway, the MAPK pathway, the Wnt pathway, vascular endothelial growth factor (VEGF), transcription factors such as FOXO1, FOXO3a and to promote cell proliferation, migration, tumour metastasis and angiogenesis in various tumours^22^,^50^. The role of AEG-1 under physiological conditions remains to be elucidated; however, it has been shown that AEG-1 is overexpressed in multiple cancers, such as oesophageal squamous cell carcinoma, breast carcinoma, melanoma, HCC and epithelial ovarian cancer^22^. AEG-1 expression was correlated with poor prognosis, enhanced invasiveness of cells and multiple resistance phenotypes to drugs such as doxorubicin, paclitaxel and cisplatin in breast cancer^22^,^50^,^51^.

Serine 298 of AEG-1 that we identified in this study represents a novel TNF-α-IKKβ-dependent regulatory site of AEG-1. On the basis of our analysis and on the basis of the fact that the sequence surrounding serine 298 matches the degenerated motif for IKKβ substrates, we hypothesized that AEG-1 may be directly phosphorylated by IKKβ. Indeed, we not only observed that AEG-1 interacts with IKKβ, but also found that IKKβ phosphorylates AEG-1 *in vitro*. Together with the proteomics data showing that inhibition of IKKβ completely abolishes TNF-α-induced phosphorylation of AEG-1, these findings establish AEG-1 as a novel, direct target of IKKβ.

AEG-1 has been reported to serve as a positive regulator of the TNF-α-dependent NF-κB pathway^52^. This prompted us to analyse the role of the IKKβ-mediated serine phosphorylation of AEG-1 in TNF-α-mediated signalling. In agreement with previously published data^33^, we observed that overexpression of AEG-1 resulted in the degradation of IκBα, thus leading to the activation of NF-κB. Strikingly, we found that the mutation of serine 298 severely impaired all steps leading to the degradation of IκBα, including its phosphorylation and ubiquitination, thus indicating the importance of IKKβ-mediated AEG-1 phosphorylation on NF-κB signalling. Molecular features of AEG-1 required for NF-κB activation were studied using a series of deletion mutants^34^. Sarkar *et al.*^34^ have shown that deletion of the N-terminal nuclear localization signal (amino acids 79–91) abolished NF-κB activation downstream of AEG-1. The deletion mutant, lacking amino acids 289–528, thus lacking the regulatory serine 298, was fully active. These data implicate that the phosphorylation of AEG-1 via IKKβ may either provide an activation signal or release an inhibitory conformation of AEG-1. We also attempted to understand the sequence of events linking IKKβ-mediated phosphorylation of AEG-1 to IκBα degradation. In the cells overexpressing AEG-1, we were able to detect interaction between these two proteins, which was then abolished by the mutation of serine 298. IκBα contains no classical domain that would provide an interface for interaction with phosphorylated serine residues^53^. Our data are therefore another clue hinting at possible phosphorylation-dependent conformational changes in AEG-1 required for interaction and subsequent degradation of IκBα. In addition to its role in IκBα degradation, AEG-1 is known to interact with the p65 subunit of NF-κB and it has been shown that the p65-AEG-1 complex translocates to the nucleus and is located at the consensus NF-κB-binding elements of various genes including the IL-8 promoter. Interestingly, we observed that mutated AEG-1, although it translocated to the nucleus, on stimulation with TNF-α, had lost its ability to interact with p65. The chromatin-bound fraction of AEG-1 was significantly smaller when serine 298 was mutated to alanine. These data show that IKKβ-mediated phosphorylation of AEG-1 is crucial for multiple steps of TNF-α-mediated gene expression, including the degradation of IκBα and p65-mediated activation of gene transcription, but not for translocation of p65 and AEG-1 to the nucleus. In accordance with these data, we also found that the mutation of serine 298 severely impaired gene expression downstream of NF-κB in various cellular systems. Along this line, we also observed that the mutation impaired AEG-1-mediated cell proliferation and survival, again pointing to a pivotal role of the IKKβ-mediated phosphorylation of serine 298 in NF-κB-dependent cellular processes.

TNF-α was proposed to mediate the cell motility of various cell types. There are, however, various and sometimes conflicting mechanisms proposed to explain these processes^54^,^55^. One of the possibilities is that TNF-α stimulation results in the expression of cell motility-relevant genes. We found that the expression of the S298A mutant of AEG-1 partially decreased the motility of MCF-7 cells, compared with wild-type AEG-1, suggesting that AEG-1 may be directly, or indirectly, through NF-κB-mediated gene expression, involved in TNF-α-induced cell motility.

TNF-α is also known to induce apoptosis of tumour cells; however, this process seems to be NF-κB-independent^56^. Since AEG-1 seems to be strongly involved in the NF-κB signalling branch of the TNF-α pathway, it is not surprising that we did not find any involvement of AEG-1 phosphorylation in TNF-α-mediated apoptosis.

AEG-1 overexpression was correlated with bad prognosis in various types of cancer. Our data suggest that the specific phosphorylation of AEG-1 can be an additional factor regulating its activity, possibly independently of the expression levels. To this end, we correlated AEG-1 phosphorylation levels with patient survival and observed that high levels of phosphorylated AEG-1 negatively correlated with patient survival, implicating a role of IKKβ-mediated phosphorylation in tumour development. We performed our analysis in samples with S298-phosphorylated tumours. We normalized the amount of phosphorylated AEG-1 to its individual total protein expression. Therefore, our data suggest that high phosphorylation levels of AEG-1 may be correlated with negative patient survival independently of gene overexpression. Taken together, our data demonstrate the importance of IKKβ-mediated phosphorylation of AEG-1 *in vivo*.

In summary, a direct combination of the SILAC-based quantitative phosphoproteomics with the random forest computational prediction allowed us to identify novel IKKβ substrates in breast cancer cells. On the basis of this approach, we demonstrate that a transmembrane protein AEG-1 is a novel substrate of IKKβ. IKKβ phosphorylates AEG-1 on serine 298, a step required for TNF-α-induced IκBα degradation and subsequent activation of NF-κB-dependent cellular processes.

## Methods

### Cell culture and transfection

MCF-7 cells were purchased from the German Collection of Microorganisms and Cell Cultures (Deutsche Sammlung von Mikroorganismen und Zellkulturen-DSMZ) and were cultured in RPMI-1640 medium supplemented with 10% fetal bovine serum (FBS), 1 mM sodium pyruvate, 1% nonessential amino acids and 10 ng ml^−1^ insulin^57^. For mass spectrometry studies, MCF-7 cells were cultured for at least six passages in RPMI-1640 medium (without arginine and lysine) (silantes) supplemented with 10% dialysed FBS and the appropriate isotope-labelled lysine and arginine as follows: Lys 0 and Arg 0 for *light,* Lys 4 and Arg 6 *for middle* and Lys 8 and Arg 10 for *heavy* condition. Lysine and arginine were added to a concentration of 0.028 and 0.0735, mg ml^−1^, respectively, in all SILAC-labelling conditions^58^. HEK293 cells were obtained from the American Type Culture Collection and cultured in DMEM medium supplemented with 10% FBS and 1% L-glutamine^59^. 4T1 cells were a kind gift from Sebastian Kobold (LMU Munich, Germany) and were cultured in RPMI-1640 medium supplemented with 10% FBS and 1% L-Glutamine^60^. cDNA transfection in HEK293 cells was carried out using the calcium phosphate method^59^. MCF-7 cells were transfected with cDNA by using TransIT-LT1 (MirusBIO) according to the manufacturer's instruction. HiPerfect reagent (Qiagen) was used for the siRNA transfection in MCF-7 cells. To establish the stable cells, MCF-7/4T1 cells were infected with lentiviral scramble or AEG-1 short hairpin RNA (shRNA) and the cells were selected using puromycin (2 μg ml^−1^). Stable cells were transfected with control pcDNA3.1-FLAG vector or the vector carrying wild-type/S298A AEG-1. The stable cells were selected using 400 μg ml^−1^ (MCF-7) or 250 μg ml^−1^ (4T1) G418 sulphate.

### Antibodies and reagents

The following antibodies were purchased from commercial sources: mouse monoclonal anti-AEG-1/MTDH clone 2F11C3 (Thermo Fischer Scientific, 1:2,000), rabbit monoclonal anti-IκBα, anti-Erk1/2-p44/42 MAPK, anti-P-Erk1/2-p44/42 MAPK, anti-p38 MAPK, anti-P-p38 MAPK and anti-Histone 3 (Cell Signalling Technology, 1:1,000), mouse monoclonal anti-α-Tubulin, anti-FLAG-horseradish peroxidase (HRP), anti-HA (haemagglutinin)-HRP, anti-γ-Tubulin (1:1,000), anti-FLAG-fluorescein isothiocyanate (FITC) and anti-FLAG affinity gel (Sigma-Aldrich), anti-IκBα (C-15), rabbit polyclonal anti-IKKα/β (Santa Cruz Biotechnology, 1:500) and anti-Ki-67 (Abcam, 1:100). SC-514 and MG132 were purchased from Calbiochem and used at the final concentration of 25 or 20 μM, respectively. TNF-α (PeproTech) was used at 10 ng ml^−1^. Labelled amino acids for SILAC were purchased from Silantes (Munich, Germany). G418 sulphate (PAA) was used at 400 μg ml^−1^ for MCF-7 and 250 μg ml^−1^ for 4T1 cells.

### shRNA/siRNA/primers

Human AEG-1 shRNAs (TRCN0000350650, TRCN0000322947, TRCN0000322949, TRCN0000322872 and TRCN0000151467) and mouse AEG-1 shRNAs (TRCN0000313386, TRCN0000312351, TRCN0000312352, TRCN0000312360 and TRCN0000312291 were purchased from SIGMA, siRNAs (SI00625793, SI04315605, SI05006421 and SI05006428) were purchased from QIAGEN. Reverse transcriptase–PCR (RT–PCR) primers for mouse FOS (5′-gggaggaccttacctgttcg-3′ and 5′-aggccagatgtggatgctt-3′); human FOS (5′-tagcaaaacgcatggagtgt-3′ and 5′-gcctggctcaacatgctact-3′) and human IL-8 (5′-agacagcagagcacacaagc-3′ and 5′-aggaaggctgccaagagag-3′) were synthesized by SIGMA.

### Plasmids and cloning

The pCMV6-Entry plasmid carrying MTDH/AEG-1 wild-type cDNA was purchased from Origene. Wild-type AEG-1 and the mutant AEG-1 (S298A) were generated using PCR and cloned into pcDNA3.1-FLAG. Clone identity was confirmed by sequencing. HA-tagged, wild-type and kinase dead (K44M) clones of IKKβ were a kind gift from D. Brandt (University of Marburg, Marburg, Germany); HA-tagged ubiquitin was a generous gift from Ivan Dikic (Goethe University of Frankfurt, Frankfurt, Germany)

### Protein digestion and phosphopeptide enrichment

MCF-7 cells were transfected with wild-type IKKβ or its kinase-inactive mutant (K44M) after at least six passages of SILAC labelling. After 48 h of transfection, cells were starved overnight and nontransfected cells were stimulated with SC-514 (25 μM) or TNF-α (10 ng ml^−1^) for 30 or 10 min, respectively, whereas transfected cells remained nonstimulated or were stimulated with TNF-α for 10 min. Cells were directly lysed in 4% SDS/0.1 M Tris HCL (pH=8.5), followed by sonication and boiling at 70 °C for 10 min. Lysates were clarified by centrifugation (13,000*g*, 10 min) and protein concentration was determined using DC assay (Bio-Rad). Approximately 3 mg protein of each labelling condition (Light—Arg0, Lys0; Middle—Arg8, Lys4; Heavy—Arg10, Lys8) were pooled and reduced using 100 mM dithiothreitol (Sigma-Aldrich) for 10 min at 56 °C and then subjected to the FASP digestion technique^61^. Briefly, samples were washed with 8 M urea, alkylated with 550 mM iodoacetamide (Sigma-Aldrich) and digested overnight in 20 mM ammoniumbicarbonate/trypsin (Promega), at an enzyme-to-protein ratio of 1:100. Peptides were collected by multiple washing of filter units, acidified to pH=2.67 with trifluoroacetic acid (TFA) and loaded on a ResourceS 1 ml SCX column (Äkta Purifier, GE Healthcare). Flow-through was collected and peptides were separated according to their charge in acidic conditions using a linear increase in salt concentration in a binary buffer system: buffer A 7 mM KH_2_PO_4_ in 30% acetonitrile (ACN) (pH=2.65) and B 7 mM KH_2_PO_4_, 350 mM KCl in 30% ACN (pH=2.65). All fractions were pooled conducting absorbance at 280 nm to a total of 8–10 fractions, concentrated and adjusted to binding conditions for Titan sphere (TiO_2_) bead-based extraction of phosphorylated peptides (80% acetonitrile, 6% TFA). Fractions were incubated twice with 2.5 mg of TiO_2_ beads and flow-throughs were incubated three times with 5 mg of TiO_2_ beads (SLSC Science). Beads were washed several– times with decreasing content of TFA (6–3%) and loaded on C8 material-containing tips. Peptides were eluted with 40% ammonia/acetonitrile (pH=11.6), concentrated in a speed vac at room temperature to almost complete dryness and diluted in acidified (0.1% formic acid or 0.5% acetic acid) H_2_O before mass spectrometry. All experiments were at least performed in duplicates.

### Liquid chromatography and mass spectrometry

Instrumentation for LC-MS/MS analysis consisted either of a NanoLC 1000 coupled via a nano-electroionization source to the quadrupole-based QExactive^62^ benchtop mass spectrometer or NanoLC and LTQ Velos mass spectrometer (Thermo Scientific). Since settings vary between instrumentation set-ups, values for the LC-LTQ Velos set-up are given in brackets. Multistage activation collision induced dissociation (MSA CID) was used for the Velos instrument to obtain *pseudo* MS^3^ in the CID fragmentation mode^63^.

Peptide separation was carried out according to their hydrophobicity on an in-house packed 50 cm (20 cm) column with 1.7 μm (3.0 μm) C18 beads (Dr Maisch GmbH) using a binary buffer system consisting of solution A: 0.1% formic acid (0.5% acetic acid) and B: 80% acetonitrile, 0.1% formic acid (80% acetonitrile, 0.5% acetic acid). Linear gradients from 7–38% B in 150–240 min were applied with a following increase to 80% B within 5 min and a re-equilibration to 5% B.

*QExactive settings:* MS spectra were acquired using 1E6 as an AGC target, a maximal injection time of 20 ms and a 70,000 resolution at 200 mz^−1^. A Top10 method was applied for subsequent acquisition of higher-energy collisional dissociation (HCD) fragmentation MS/MS spectra of the top 10 most intense peaks. Resolution for MS/MS spectra was set to 35,000 at 200 mz^−1^, AGC target to 5E5, max injection time to 120 ms and the isolation window to 1.3 Th.

*Velos settings*: the resolution for MS spectra was set to 30,000 at 400 mz^−1^ after accumulation of 1E6 ions (AGC target) within a maximal injection time of 60 ms. Top 15 method was applied for MSA MS/MS spectra at a resolution of 7,500 at 200 mz^−1^. AGC target and maximal injection time were set to 1E4 and 30 ms, respectively. CID fragmentation was carried out with multistage activation and normalized collision energy was set to 35.

### Data analysis

More than 120 raw files from all four experimental set-ups in at least duplicates were processed using MaxQuant (1.4.1.2)^64^ and the implemented Andromeda search engine^65^. For protein assignment, electrospray ionization-tandem mass spectrometry (ESI-MS/MS) fragmentation spectra were correlated with the Uniprot human database (v. 2014) including a list of common contaminants. Searches were performed with tryptic specifications and default settings for mass tolerances for MS and MS/MS spectra. Carbamidomethyl at cysteine residues was set as a fixed modification, while oxidation at methionine, acetylation at the N terminus and phosphorylation of serine, threonine and tyrosine (STY) were defined as variable modifications. The minimal peptide length was set to seven amino acids, and the false discovery rate for proteins and peptide-spectrum matches to 1%. A minimal ratio count for SILAC pairs was required and we enabled the match-between-run feature with a time window of 1 min. The minimal score for modified peptides was set to 0 (default 40). Before analysis, the data set was filtered to remove contaminants and reverse entries. Classification of phosphorylation sites into three classes depending on the localization probability and delta score calculated using MaxQuant is specified in the main text.

### Random forest-based prediction of potential IKKβ substrates

To perform Random forest analysis to predict novel IKKβ substrates, we first manually filtered known TNF-α/IKKβ-dependent phosphorylation sites. In total, we found 11 IKKβ and 25 TNF-α targets, which represent the positive data sets. Conversely, the negative data set was created by resampling of the remaining phosphorylation sites, which were quantified in all experiments (>10,000; Supplementary Fig. 3a). The classifiers were trained using numerous features (i) H/L, M/L and H/M SILAC ratios from all four experimental set-ups (Fig. 1a), (ii) sequence scoring matrix surrounding the phosphorylation site (31 residues), (iii) capability of overexpressed IKKβ to phosphorylate the specific site without TNF-α stimulation, (iv) effect of overexpressed IKKβ mutant compared with wild-type with TNF-α stimulation (TNF-α+IKK/TNF-α+mutant) and (v) inhibitory effect of mutant IKKβ (TNF-α+MUT/TNF-α; Supplementary Fig. 3b). Random forest is an ensemble-learning algorithm, which minimizes information loss using downsampling of the dominant class. Here we allowed the dominant group to be four times the size of the positive class (validated substrates), retaining sensitivity for the positive hits. The caret package in the statistical environment R was used to realize construction of trees (*n*=1,500), downsampling, random forest tuning (receiver operating characteristic (ROC)-based, tune length=5, parameter tuned: mtry—no. of features used at each split) and prediction as well as evaluation and comparison with other machine-learning algorithms^66^,^67^. Although the automatically calculated out-of-the-bag error gives an unbiased estimate of the error, we performed a ROC curve analysis to assess the influence of the downsampling approach on a test data set including true positive hits, and remaining resampled data from the training data set were assigned as true negatives. False negatives and false positives are subsequently calculated. The ROC analysis revealed an increased sensitivity and specificity due to the downsampling strategy conducting the area under the curve. We obtained a value of 0.99 for downsampling versus 0.89 for using the complete negative data set at once. Trained predictors were then used to calculate a probability of each phosphosite including the positive hits to be a member of the positive class, for example, IKKβ substrate, or TNF-α-dependent. Comparison of both calculated probabilities allows for proposition of specificity.

### Immunoprecipitation and western blotting

For protein immunoprecipitation, MCF-7 or HEK293 cells were lysed in ice-cold radioimmunoprecipitation (RIPA) buffer (1% Triton X-100, 150 nM NaCl, 50 mM Tris pH 7.4, 0.1% sodium dodecyl sulfate, 0.25% sodium deoxycholate, 1 μg ml^−1^ of each leupeptin, aprotinin and pepstatin, 1 mM 4-(2-aminoethyl)-benzosulfonylfluoridhydrochloride and 1 mM Na_3_VO_4_). Protein extracts were purified with centrifugation and were incubated with the antibody for the respective protein in presence of Protein A/G PLUS Agarose (Santa Cruz Biotechnology). Anti-FLAG affinity gel was used to immunoprecipitate FLAG-tagged MTDH. Precipitated proteins were then centrifuged and washed several times using ice-cold RIPA buffer. Finally, antibody–antigen complexes were eluted by boiling in Laemmli buffer and samples were subjected to western blotting. To determine the phosphorylation of Erk1/2 MAPK, p38 MAPK or IκBα degradation, MCF-7 cells were transfected with wild-type or mutated (K44M) IKKβ. Forty-eight hours after transfection, cells were starved for 12 h and incubated with SC-514 (25 μM) and/or TNF-α (10 ng ml^−1^). Cells were lysed and lysates were subjected to western blot analysis as described above. To determine AEG-1 phosphorylation of Erk1/2 and IκBα degradation, MCF-7 cells were transfected with siRNA targeting AEG-1 or HEK293 cells transfected with wild-type/S298A AEG-1. After 48 h, cells were starved, stimulated with control or TNF-α (10 ng ml^−1^) for 10 min and lysed. For subcellular localization, proteins were fractionated using the Subcellular Protein Fractionation Kit for Cultured Cells (Thermo). Cell lysates, immunoprecipitates and fractions were separated using SDS–PAGE and transferred to a nitrocellulose membrane. Proteins were detected using their respective antibodies and visualized using enhanced chemiluminescence system (GE Healthcare and Millipore). Full-size western blot images are presented in Supplementary Figs 6–11.

### Radioactive *in vitro* kinase assay

To visualize protein AEG-1 phosphorylation *in vitro,* FLAG-tagged wild-type or S298A protein AEG-1 was transfected into HEK293 cells. After 48 h, cells were starved overnight and protein AEG-1 was immunoprecipitated using anti-FLAG affinity gel (Sigma-Aldrich). Purified recombinant IKKβ (ProQinase GmbH) was incubated with immunoprecipitate in the presence of 2 × kinase buffer, 10 μM cold ATP (Cell Signalling), 5 μCi γ^32^-ATP (Hartmann Analytic GmbH) for 30 min at 30 °C. The reaction was stopped by adding 4 × Laemmli buffer and was subjected to boiling for 5 min. The samples were separated using SDS–PAGE gel and the gel was dried and exposed to an X-ray film (GE Healthcare).

### Immunofluorescence and flow cytometry

For immunostaining, MCF-7 cells were either transfected with siRNA targeting AEG-1 or FLAG-tagged wild-type/S298A AEG-1 cDNA and were incubated for 48 h. After serum starvation overnight, cells were stimulated with control buffer or TNF-α (10 ng ml^−1^) for 10 min and fixed in 4% paraformaldehyde (PFA) for 15 min at room temperature. Fixed cells were permeabilized by 0.5% triton and immunostained using anti-AEG-1 or anti-FLAG-FITC for endogenous AEG-1 or overexpressed AEG-1, respectively. Anti-IκBα was used for counterstaining. Nuclei were stained with Hoechst 33342 (Invitrogen) and imaged under epifluorescent microscope.

For proliferation assay using flow cytometry (FACS), serum-starved FLAG-tagged wild-type/S298A AEG-1 cDNA-transfected HEK293 cells were treated with TNF-α (10 ng ml^−1^) for 12 h. Cells were then trypsinized and the cell pellet was fixed in 4% PFA, permeabilized by 0.5% triton and stained using anti-FLAG-FITC. Cells were counterstained with anti-Ki-67. Ki-67-positive cells were quantified in the sorted FLAG-positive stained cells in BD FACS Canto II. For cell cycle analysis, MCF-7 and 4T1 cells stably expressing scrambled shRNA or shRNA against AEG-1 together with FLAG-tagged wild-type or S298A AEG-1 were serum-starved, treated with control or TNF-α (10 ng ml^−1^) and trypsinized. Cells were then pelleted and the pellets were fixed in 70% ethanol. Cells were stained using 7-aminoactinomycin D (7-AAD) and cell cycle phases were analysed in BD FACS Canto II.

### Chromatin immunoprecipitation (ChIP)

MCF-7 cells were transiently transfected with wild-type or S298A AEG-1 cDNA and incubated for 48 h. Cells were then starved and stimulated with TNF-α (10 ng ml^−1^) or left untreated for 12 h. For ChIP experiments, ∼2–5 × 10^7^ cells were crosslinked with 1% formaldehyde for 10 min at room temperature. The reaction was then quenched using 0.125 M glycine for 5 min at room temperature and washed with ice-cold PBS. Cells were lysed using cell lysis buffer (5 mM HEPES pH 8.0, 85 mM KCl, 0.5% NP-40 and protease inhibitor cocktail) and nuclei were enriched for sonication. The nuclei were then sheared by sonication to an average chromatin fragment size of 200–400 bp using Biopruptor (Diagenode). Chromatin was precleared and incubated with anti-FLAG affinity gel overnight at 4 °C, and immune complexes were precipitated by protein A beads (Diagenode), which was pre-equilibrated with sonicated salmon sperm DNA and BSA. Immunoprecipitated material was then washed extensively, and the crosslinks were reversed using elution buffer (10 mM NaHCO_3_, 1% SDS) overnight at 65 °C (ref. 68). ChIP DNA from eluted chromatin was purified using the PCR purification kit (Qiagen). FOS promoter site-binding primers were 5′-gagcagttcccgtcaatcc-3′ and 5′-gcatttcgcagttcctgtct-3′. The enrichment of target DNA over input was calculated using the ΔΔ*C*_t_ method with results presented as the mean plus or minus s.e.m.

### Migration and invasion assay

MCF-7 (3 × 10^4^)/4T1 (10^4^) cells stably expressing wild-type or S298A AEG-1 on the background of stable AEG-1 knockdown were starved and seeded into either 96-well-migration upper chambers or 24-well matrigel chamber (Corning). Control buffer or TNF-α (10 ng ml^−1^) were added to the lower chamber of the respective wells. The cells were then allowed to migrate/invade for 24 h and the migrated/invaded cells at the lower surface of the filter were fixed in methanol, stained using toluidine blue and counted.

### Real-time, label-free measurement of cell proliferation

Cell proliferation was measured using the xCELLigence system (Roche), according to the manufacturer's instructions. Briefly, MCF-7 cells were transfected with wild-type or S298A protein AEG-1 cDNA. After 48 h of transfection, cells were trypsinized, seeded on E-Plate (ACEA Biosciences Inc.) and cell impedance measurement was carried out for 48 h.

### Colony formation assay

Colony formation assay was adapted from ref. 69. Cells were trypsinized and 100 cells per well were seeded onto six-well plates. After 5 days, cells were fixed and stained using toluidine blue. Colonies that contained more than 50 cells were counted and percentage of colony formation was determined by the following formula:


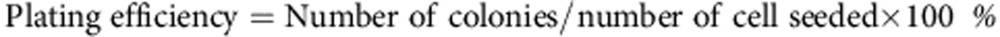


### Statistical analysis

Comparison of two groups was carried out by a two-sided *t*-test. To identify significantly regulated phosphorylation sites, the FDR was controlled (multiple testing correction) using a permutation-based algorithm, adapted from ref. 70 in the statistical environment R using the siggenes package. Fudge factor s0 was set to 0.6, and 500 permutations were used for FDR estimation. All data are provided in Supplementary Data 1, indicating significance of fold change after TNF-α stimulation compared with the basal level. In general, data are represented as mean±s.e.m., except for SILAC ratios, where the median and s.d. are given. The experiments were not randomized and the investigators were not blinded to allocation during experiments and outcome assessment. Except for mass spectrometry data, all data shown are mean±s.d. *P<0.05.

### Patient survival analysis

Proteomics and phosphoproteomics data from a clinical study^42^ were downloaded from the Clinical Proteomic Tumor Analysis Consortium (NCI/NIH) website (CPTAC Data Portal: https://cptac-data-portal.georgetown.edu/cptac/ Data set S020). To check for the impact of AEG-1 phosphorylation, we utilized the iTRAQ-based phosphoproteome and proteome report files, analysed by the CDAP pipeline. First, we filtered for patients with AEG-1 S298 phosphorylation. Next, we extracted AEG-1 protein and phosphoprotein (only those containing phosphorylated serine 298) expression values for the filtered patients and mapped the expression values on the clinical data provided by CPTAC. Phosphoprotein expression was normalized to protein expression. We used both ends of the AEG-1 phosphorylation expression distribution (top 20 versus lowest 20) for a survival analysis based on the clinical data (vital status, days to last follow-up). To access statistical significance, we performed a log-rank test. Curves were plotted using GraphPad Prism v 5.04.

## Author contributions

J.M.S., M.K., R.K.K. and H.N designed experiments and analysed results. H.N. and R.K.K. performed peptide enrichment. H.N. performed mass spectrometry-based measurement of phosphopeptides as well as machine-learning for computational prediction. R.K.K. performed molecular cloning and mutagenesis of AEG-1, together with biochemical and biological analyses. H.K. performed FACS analysis, sorting and quantified gene expression using real-time PCR and K.S. performed ChIP assay. M.L. analysed patients' survival. T.S. performed migration and invasion assays. J.M.S., H.N., M.K. and R.K.K. wrote the manuscript.

## Additional information

**Accession codes.** The mass spectrometry proteomics data have been deposited to the ProteomeXchange Consortium via the PRIDE partner repository with the dataset identifier PXD001873.

**How to cite this article:** Krishnan, R. K. *et al.* Quantitative analysis of the TNF-α-induced phosphoproteome reveals AEG-1/MTDH/LYRIC as an IKKβ substrate. *Nat. Commun.* 6:6658 doi: 10.1038/ncomms7658 (2015).

## Supplementary Material

Supplementary Figures Supplementary Figures 1-11

Supplementary Data 1 Identified phosphosites in the complete dataset (Experiment 1-4). Sites used for training the classifiers are indicated. Description of each column is inside the file.

Supplementary Data 2 Identified protein groups by acquired MS raw data and processing by MaxQuant. Description of each column is inside the file.

Supplementary Data 3 Phosphorylation sites that were quantified with 13 SILAC ratios in the 4 experiments including the training data set. The classifiers were used to determine a scores for IKKβ and TNF-a dependent sites. This table is related to Fig. 1e and Supplementary Fig. 3. Description of each column is inside the file.

## Figures and Tables

**Table 1:** Potential phosphorylation targets regulated in the TNF-α/IKKβ-mediated pathway.

**Protein name**	**Gene name**	**Phosphorylation site**	**Uniprot ID**	**IKKβ score**	**TNF-α score**	**Sequence window**
TOM1-like protein 2	*TOM1L2*	457	Q6ZVM7	87.8	92.73	DLEEGVT(pS)EEFDKFLE
Receptor-interacting serine/threonine protein kinase 1	*RIPK1*	320	Q13546	84.26	81.2	AVVKRMQ(pS)LQLDCVAV
Astrocyte-elevated gene-1	*AEG-1*	298	Q86UE4	83.06	76.2	VKLSSQI(pS)AGEEKWNS
Autophagy-related protein 2 homologue B	*ATG2B*	497	Q96BY7	82.33	80.33	SLPSRSV(pS)VDESRPEL
Synaptotagmin-like protein 2	*SYTL2*	649	Q9HCH5–8	82.13	66.93	PHFYRAA(pS)QTSEMKDK

IKKβ, I-kappa-B kinase; TNF, tumour necrosis factor.
