# Supplementary material for: Quantitative analysis of the TNF-α-induced phosphoproteome reveals AEG-1/MTDH/LYRIC as an IKKβ substrate
Source: Nat Commun. 2015 Apr 7;6:6658. doi: 10.1038/ncomms7658 (PMC4396366; doi:10.1038/ncomms7658)
Supplement: Supplementary Figures — 1-11 [file ncomms7658-s1.pdf]

**Supplementary Figure 1**

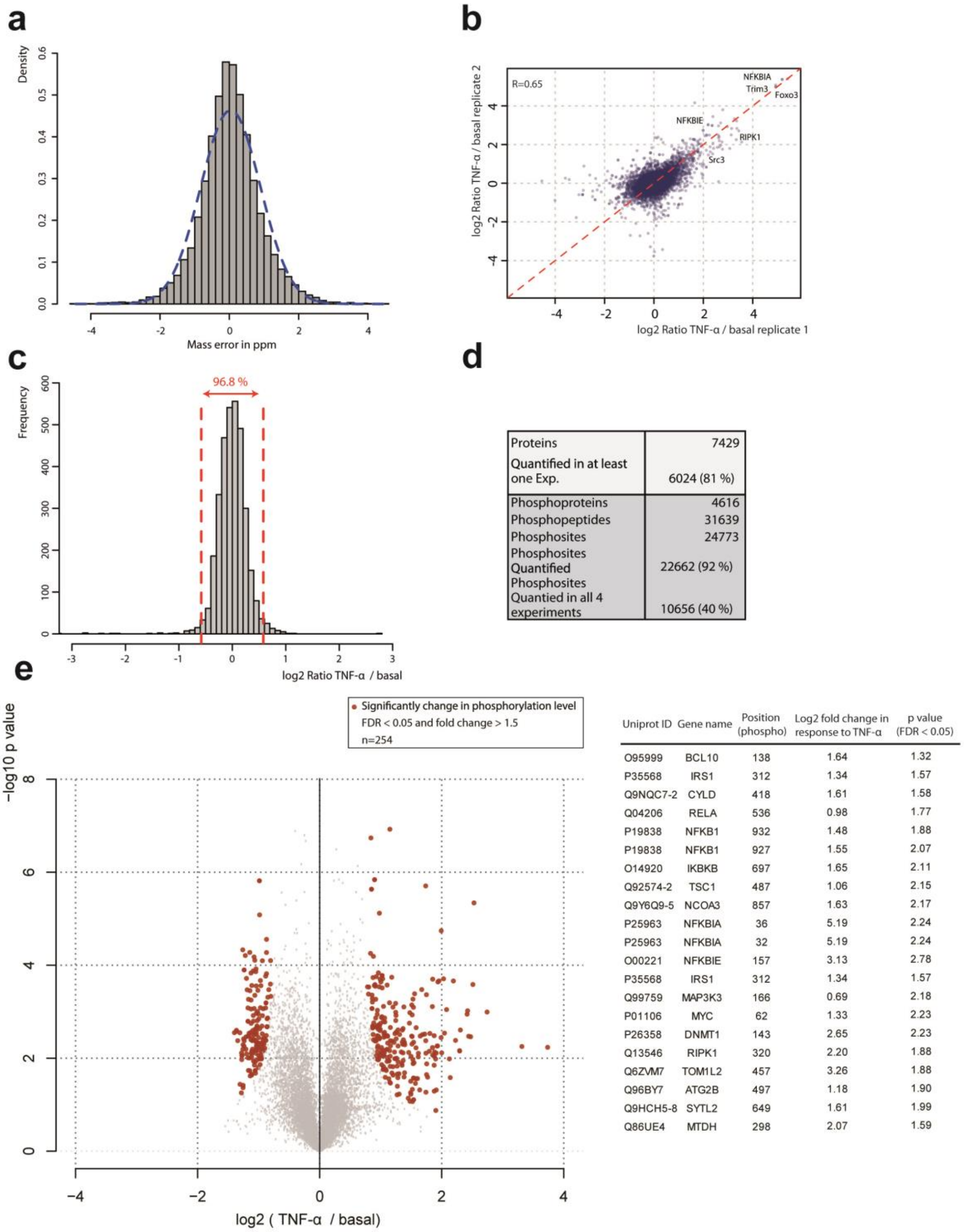


| | |
|---|---|
| Proteins | 7429 |
| Quantified in at least one Exp. | 6024 (81 %) |
| Phosphoproteins | 4616 |
| Phosphopeptides | 31639 |
| Phosphosites | 24773 |
| Phosphosites Quantified | 22662 (92 %) |
| Phosphosites Quantied in all 4 experiments | 10656 (40 %) |

| Uniprot ID | Gene name | Position (phospho) | Log2 fold change in response to TNF-α | p value (FDR < 0.05) |
|---|---|---|---|---|
| O95999 | BCL10 | 138 | 1.64 | 1.32 |
| P35568 | IRS1 | 312 | 1.34 | 1.57 |
| Q9NQC7-2 | CYLD | 418 | 1.61 | 1.58 |
| Q04206 | RELA | 536 | 0.98 | 1.77 |
| P19838 | NFKB1 | 932 | 1.48 | 1.88 |
| P19838 | NFKB1 | 927 | 1.55 | 2.07 |
| O14920 | IKBKB | 697 | 1.65 | 2.11 |
| Q92574-2 | TSC1 | 487 | 1.06 | 2.15 |
| Q9Y6Q9-5 | NCOA3 | 857 | 1.63 | 2.17 |
| P25963 | NFKBIA | 36 | 5.19 | 2.24 |
| P25963 | NFKBIA | 32 | 5.19 | 2.24 |
| O00221 | NFKBIE | 157 | 3.13 | 2.78 |
| P35568 | IRS1 | 312 | 1.34 | 1.57 |
| Q99759 | MAP3K3 | 166 | 0.69 | 2.18 |
| P01106 | MYC | 62 | 1.33 | 2.23 |
| P26358 | DNMT1 | 143 | 2.65 | 2.23 |
| Q13546 | RIPK1 | 320 | 2.20 | 1.88 |
| Q6ZVM7 | TOM1L2 | 457 | 3.26 | 1.88 |
| Q96BY7 | ATG2B | 497 | 1.18 | 1.90 |
| Q9HCH5-8 | SYTL2 | 649 | 1.61 | 1.99 |
| Q86UE4 | MTDH | 298 | 2.07 | 1.59 |

**Supplementary Figure 1: Statistical analysis of the phosphoproteome and proteome dataset.** (a) Histogram indicating the mass errors of detected phosphorylation sites and 95 % of all detected phosphopeptides fit less than 2 ppm. Blue broken-line shows Gaussian distribution. (b) Correlation between the biological replicates was determined by Pearson

correlation coefficient. Known phosphoproteins involved in the TNF-α-mediated signalling pathway are indicated. (c) Expression changes were analysed from the identified proteins and 96.8 % of detected proteins did not show changes in protein expression levels upon TNF-α stimulation. (d) Number of identified and quantified proteins and phosphopeptides from the global phosphoproteomics screen are represented in the table. (e) Volcano plot showing the fold change in response to TNF-α stimulation compared to p- values from a two-sided t-test. Red dots represent significant changes at a FDR cut off of 0.05 (parameters: s0=0.6, permutations: 500). The table depicts selected candidates for up-regulated phosphorylation sites in response to TNF-α stimulation.

## Supplementary Figure 2

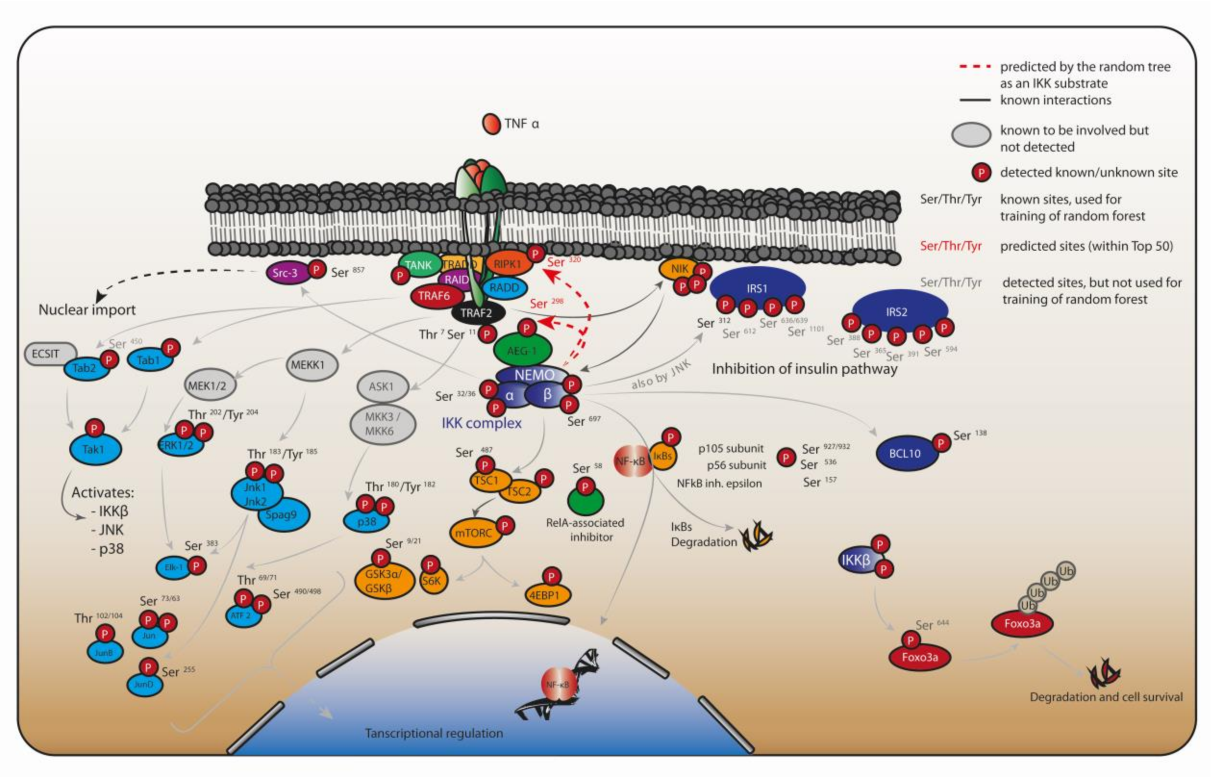


**Supplementary Figure 2: TNF-α phosphoproteomic network.** Indicated are the known substrates of IKKβ as well as the activated proteins upon TNF-α stimulation. Novel potential IKKβ substrates were shown in red arrows. Identified phosphorylation sites / phosphoproteins which were not included in random forest analysis were represented in pale grey.

## Supplementary Figure 3

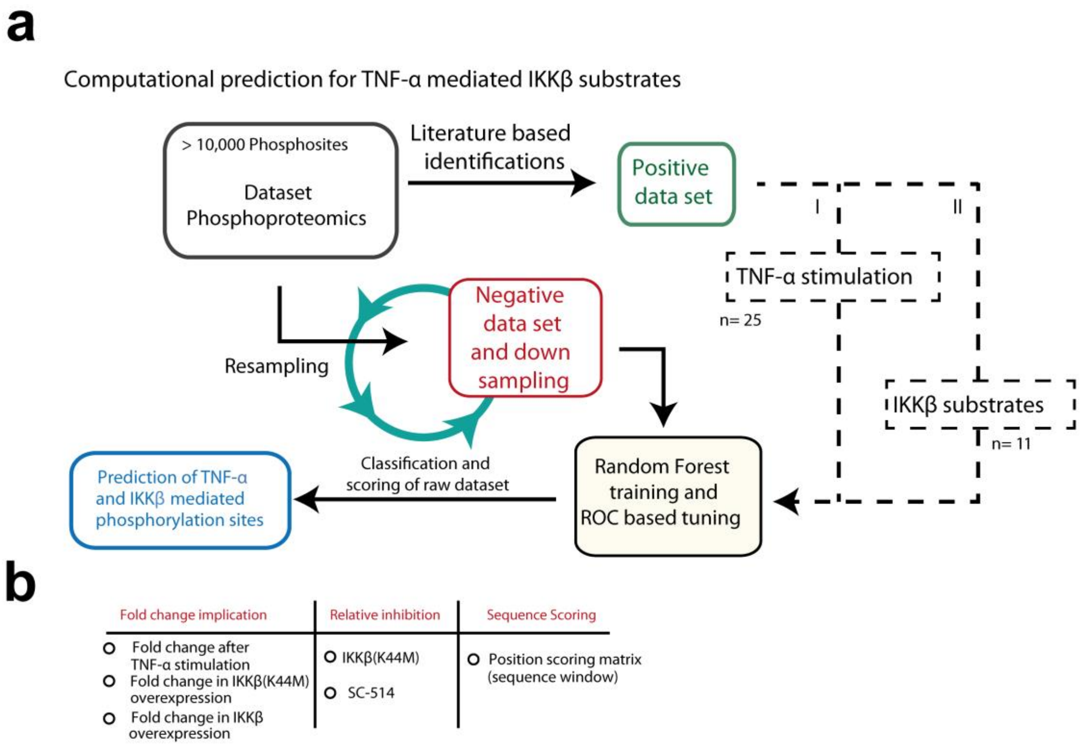


**Supplementary Figure 3: Schematic overview of computational workflow.** (a) Random forest approach with down sampling was used for the prediction of IKKβ substrates and TNF-α mediated phosphorylation sites. Positive phosphorylation sites were identified by literature search that were utilized to train the classifiers. Resampling of the remaining dataset was practiced to create a negative data set. Both classifiers were then utilized to predict the substrates of IKKβ and TNF-α dependent phosphorylation sites. (b) Table representing features of each phosphorylation site used. Sequence scoring matrix was applied for the assessment of amino acid based identification of the IKKβ substrates.

**Supplementary Figure 4**

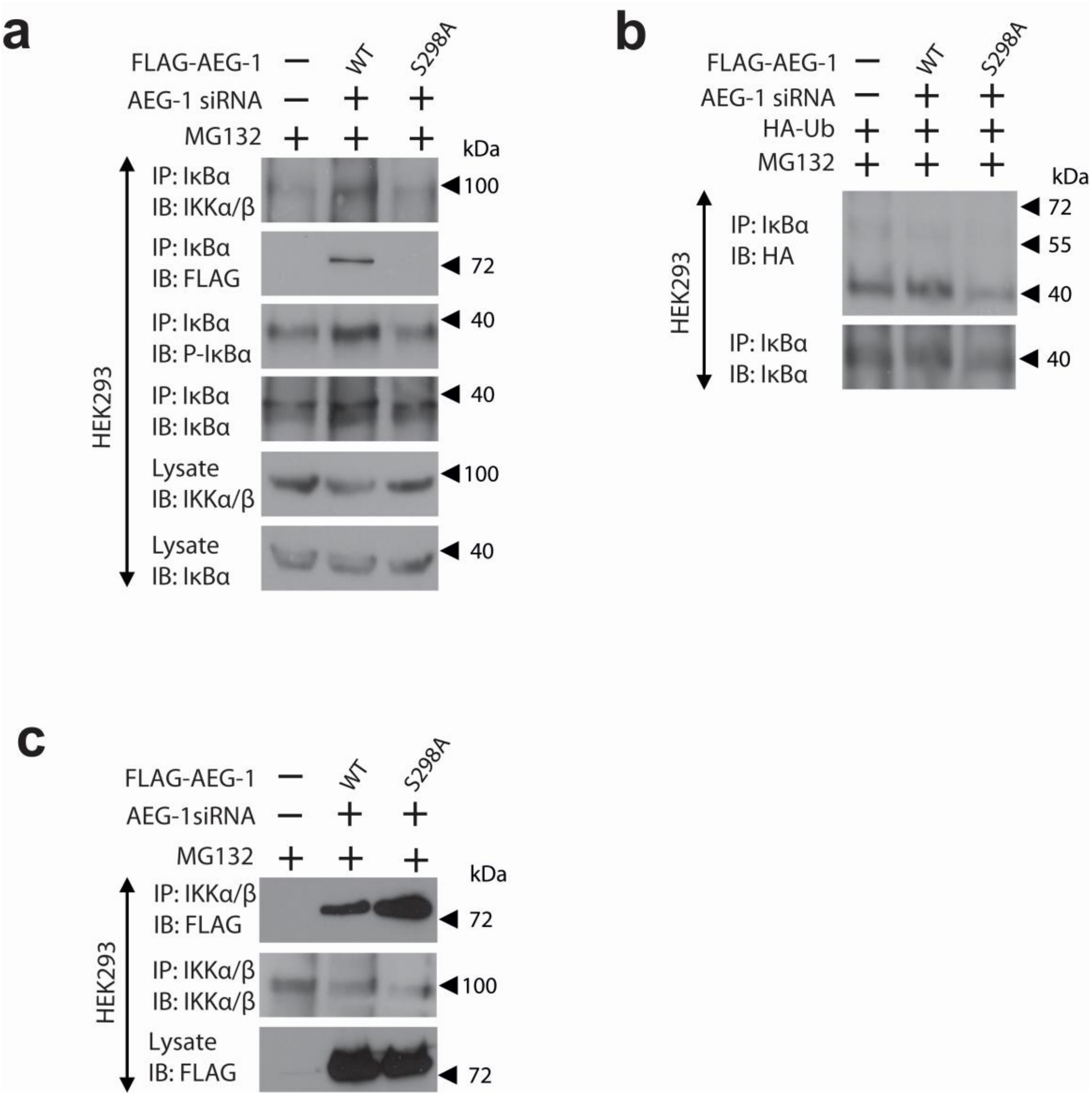


**Supplementary Figure 4: Phosphorylation of AEG-1 serine 298 is required for IKKβ-mediated IκBα phosphorylation, ubiquitination and degradation.** (a) HEK293 cells were transfected with control or AEG-1 siRNA. After 24 hours, the cells were transfected with either vector or FLAG-tagged wild-type (WT) or S298A mutant of AEG-1. 48 hours later, cells were treated with MG132 (20µM) for 2 hours and lysed. IκBα was immunoprecipitated from cell lysates. The precipitates were immunoblotted using respective antibodies (b) HEK293 cells were transfected as in (a) in combination with HA-tagged ubiquitin. IκBα was immunoprecipitated as described above. The precipitates were immunoblotted using anti-HA antibody. (c) HEK293 cells were treated as above. Protein complex from the precipitates were immunoblotted using anti-FLAG and anti IKKα/β antibodies.

**Supplementary Figure 5**

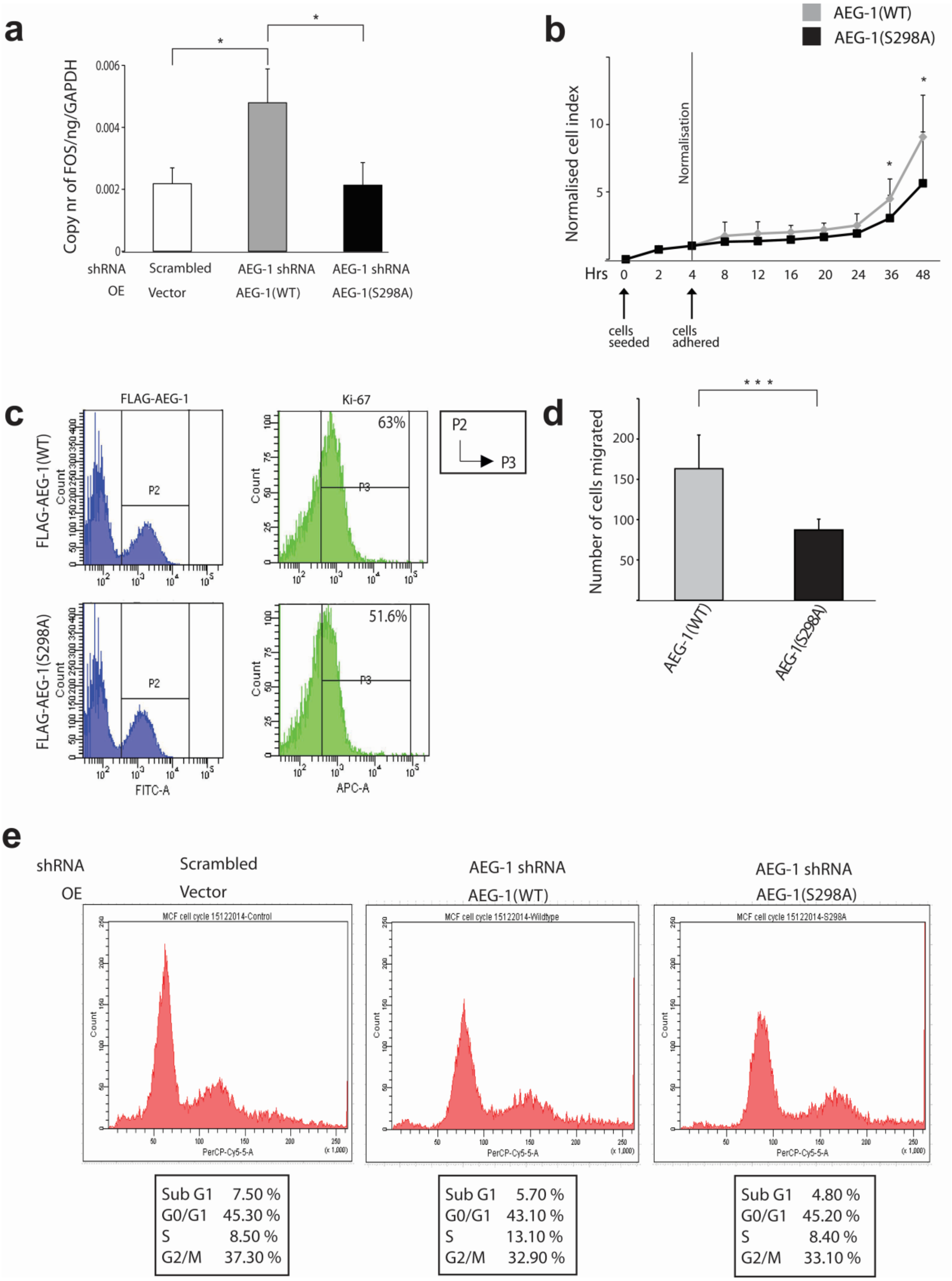


**Supplementary Figure 5: Role of serine 298 in NF-κB-mediated gene expression, cell proliferation and migration**. (a) RNA from stable 4T1 cells was isolated and expression of NF-κB-regulated Fos gene was determined (b) MCF-7 cells transfected with wild-type (WT)

or mutant (S298A) AEG-1 were seeded onto the E-Plate. Real-time, label-free impedance measurement was carried out for 48 hours. (c) Wild-type or S298A AEG-1 was overexpressed in HEK293 cells. After starvation and stimulation with TNF-α for 12 hours, cell pellets were collected, fixed with 4% PFA and immunostained using anti-FLAG-FITC and anti-Ki-67. The percentage of Ki-67-positive cells in the FLAG-positive group was quantified. (d) MCF-7 cells transfected with wild-type or S298A AEG-1 were trypsinized and counted. $3\times10^5$ cells in RPMI containing 0.5% FBS were seeded into the upper chamber of a transwell plate. Cells were allowed to migrate for 24 hours. Migrated cells were then fixed, stained and counted as described in Methods. (e) Cell cycle distributions of 7-AAD stained stable MCF-7 cells. All plots represent mean values -/+ SD from triplicates. *, $P < 0.05$.

**Supplementary Figure 6**

Original immunoblot data for Fig. 1c

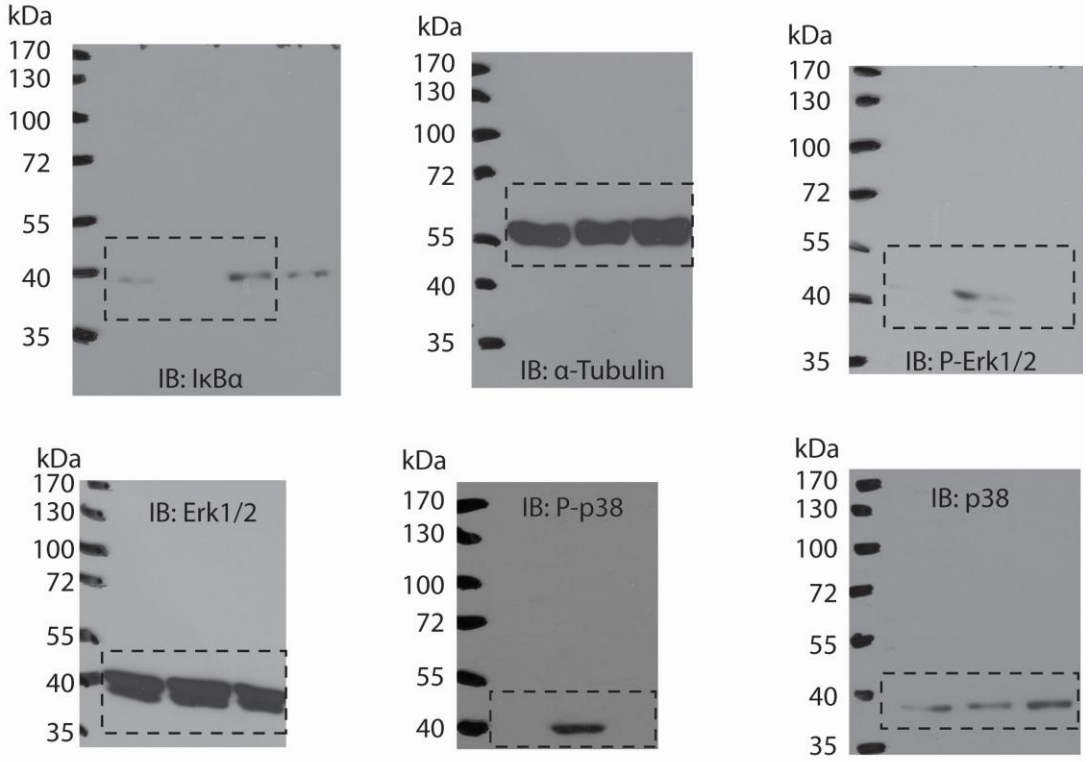


Original immunoblot data for Fig. 1d

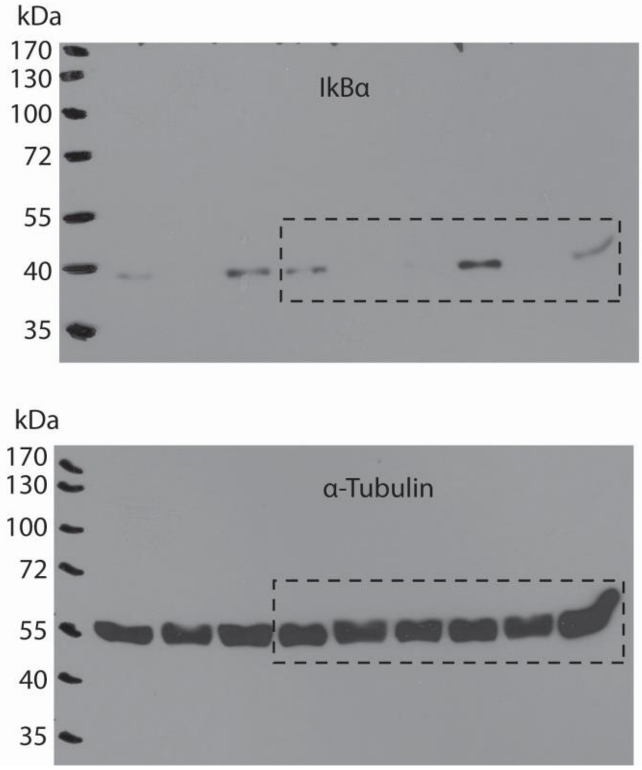


**Supplementary Figure 6: Original Western blots for Figure 1**

**Supplementary Figure 7**

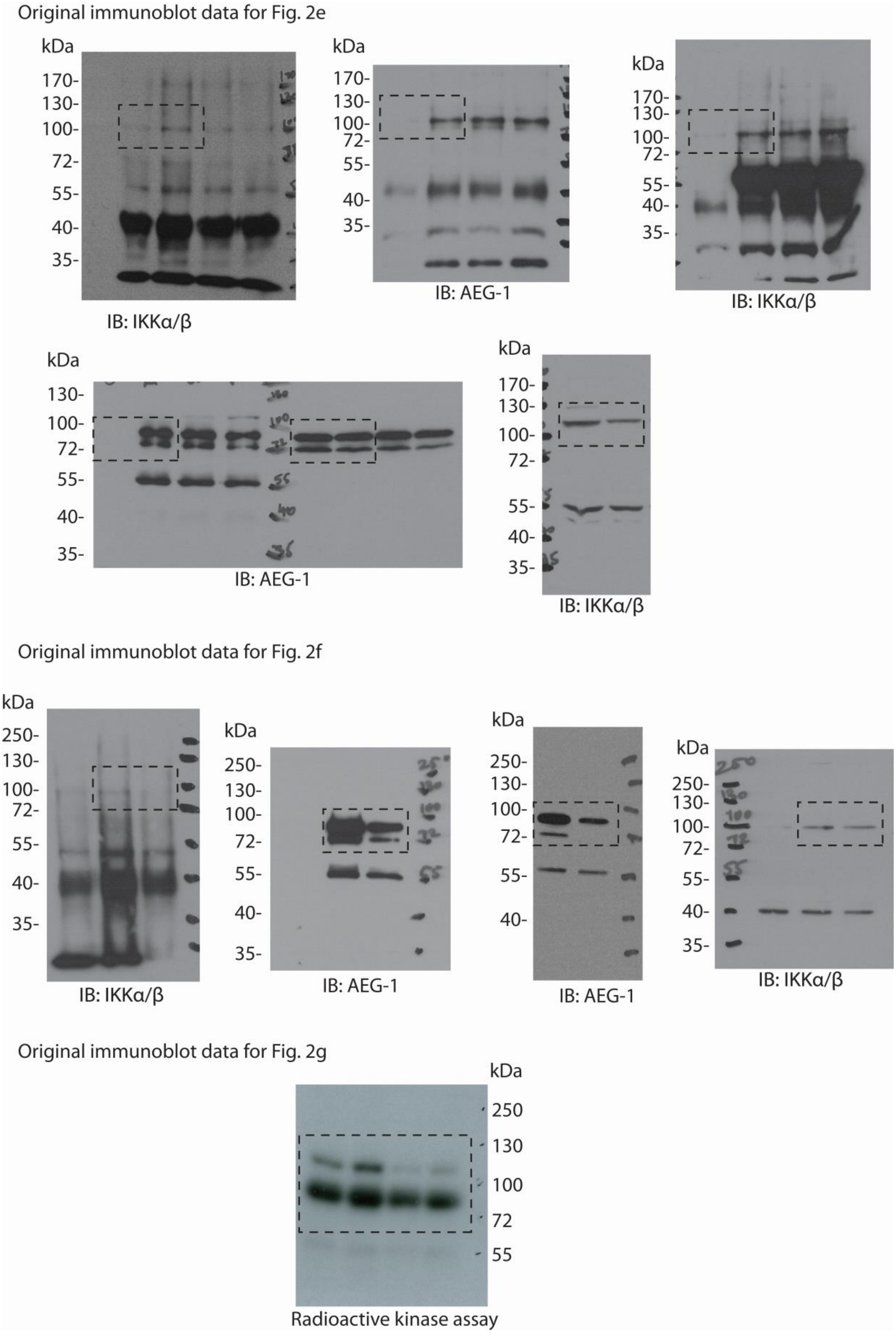


**Supplementary Figure 7: Original Western blots for Figure 2**

**Supplementary Figure 8**

Original immunoblot data for Fig. 3e

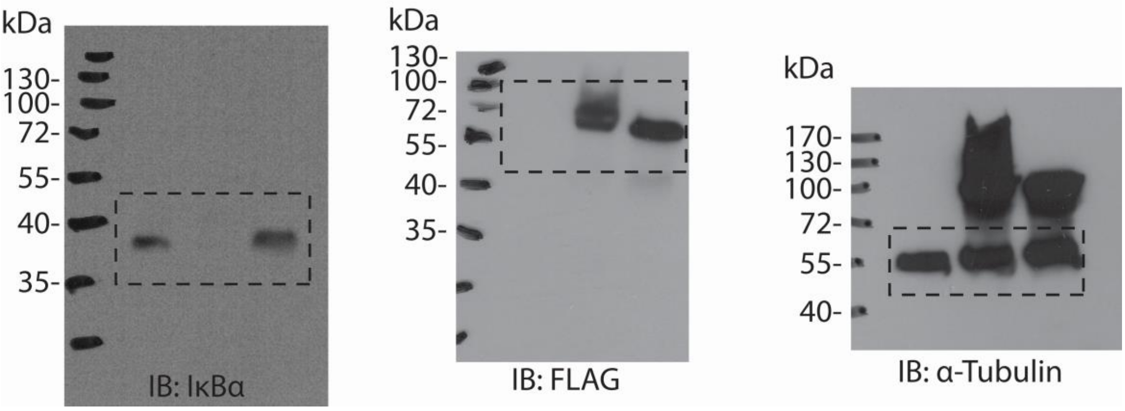


**Supplementary Figure 8: Original Western blots for Figure 3**

**Supplementary Figure 9**

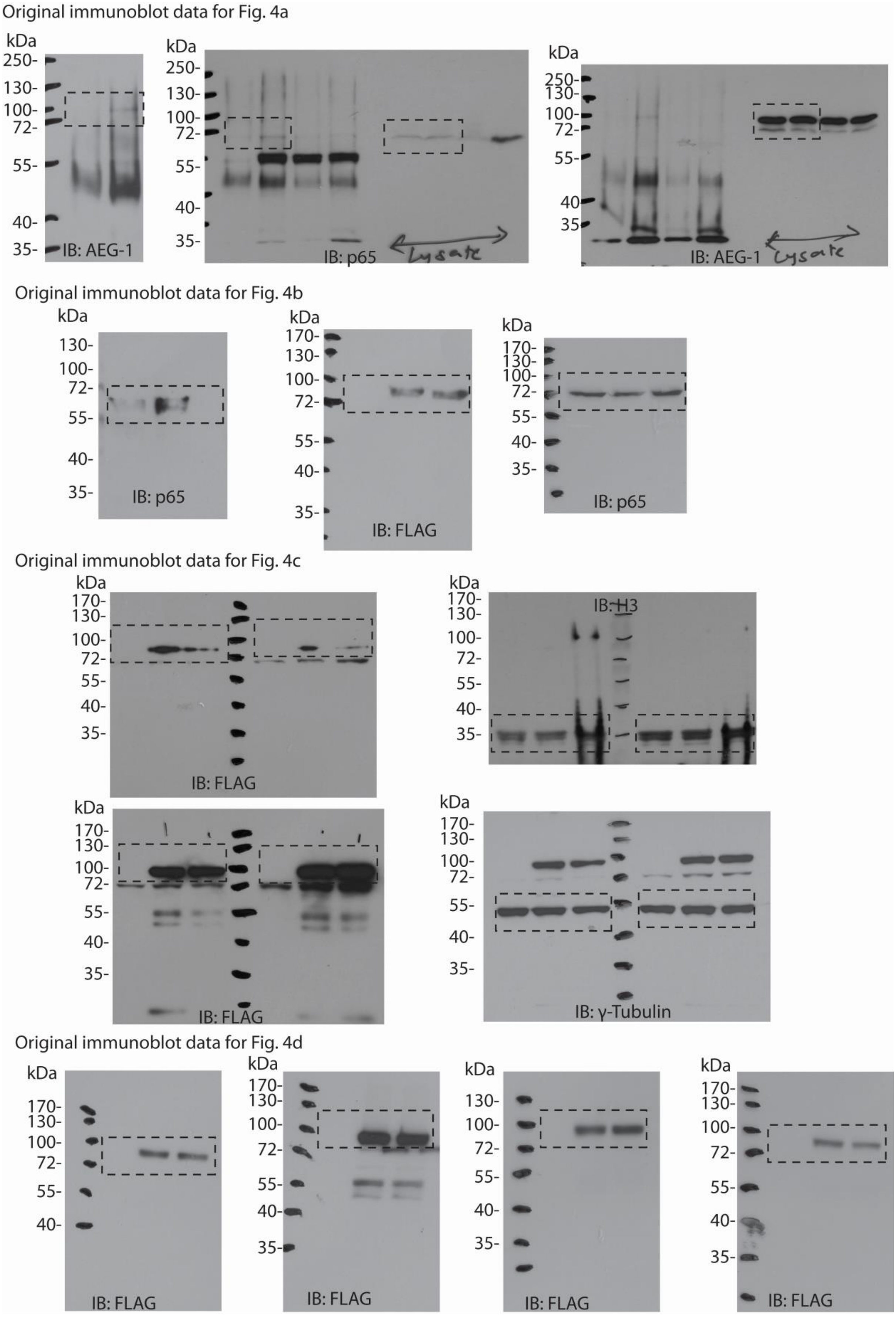


**Supplementary Figure 9: Original Western blots for Figure 4**

**Supplementary Figure 10**

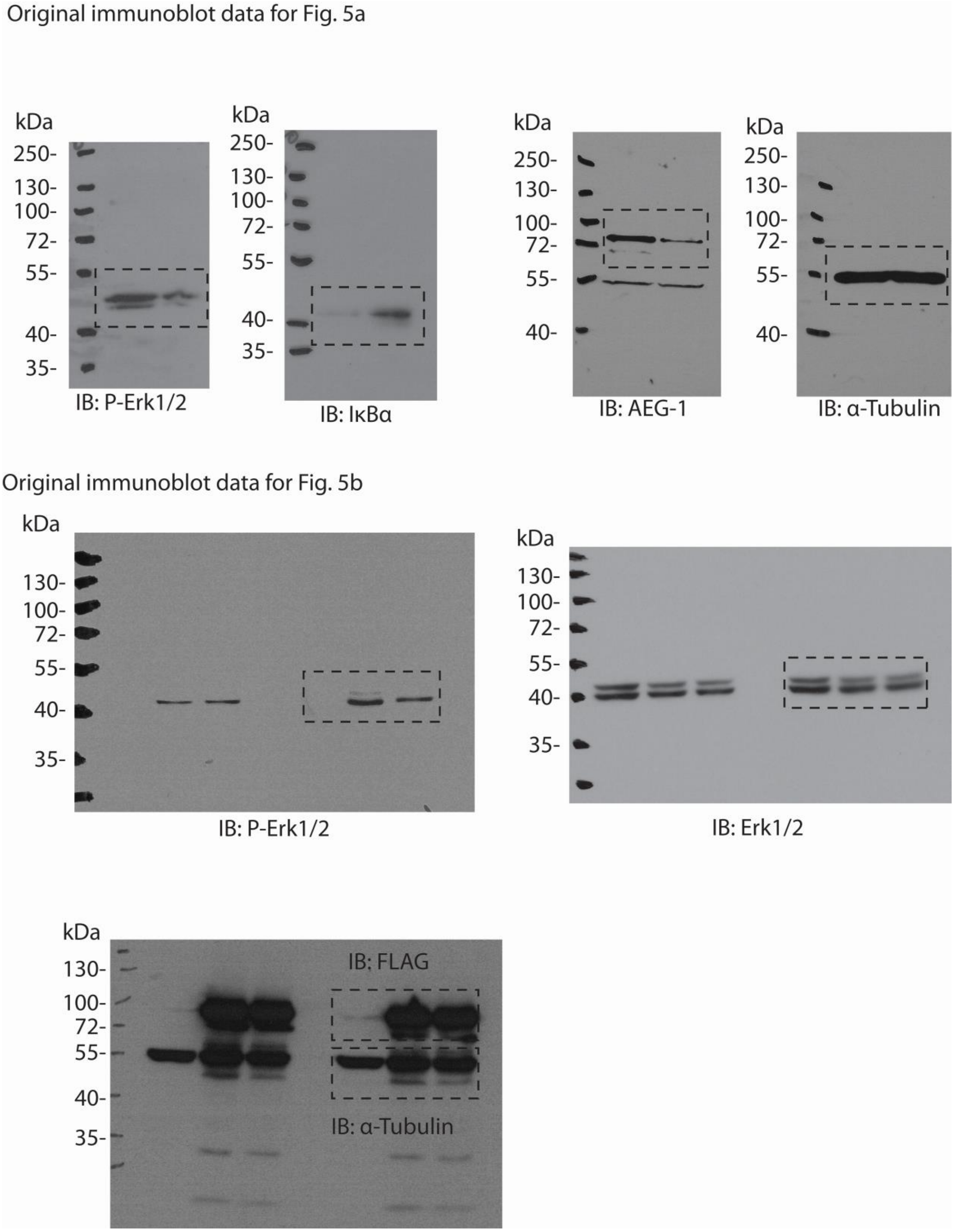


**Supplementary Figure 10: Original Western blots for Figure 5**

**Supplementary Figure 11**

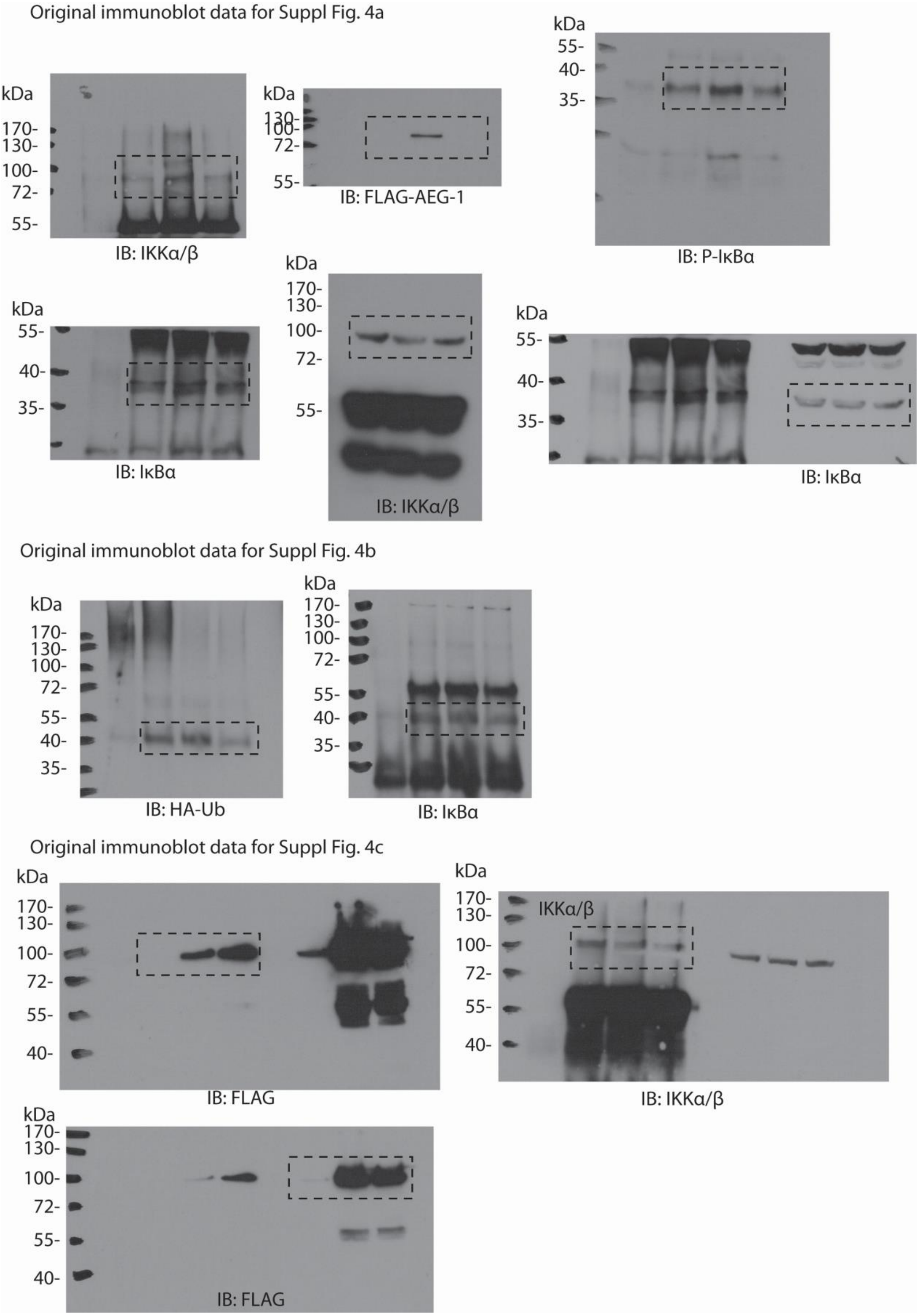


**Supplementary Figure 11: Original Western blots for Supplementary Figure 4**
